# Interhemispheric connections between olfactory bulbs improve odor detection

**DOI:** 10.1371/journal.pbio.3000701

**Published:** 2020-04-20

**Authors:** Florence Kermen, Pradeep Lal, Nicholas G. Faturos, Emre Yaksi

**Affiliations:** 1 Kavli Institute for Systems Neuroscience and Centre for Neural Computation, Faculty of Medicine and Health Sciences, Norwegian University of Science and Technology, Trondheim, Norway; 2 Neuro-Electronics Research Flanders, Leuven, Belgium; 3 Department of Biology, Faculty of Natural Sciences, Norwegian University of Science and Technology, Trondheim, Norway; University of Lausanne, SWITZERLAND

## Abstract

Interhemispheric connections enable interaction and integration of sensory information in bilaterian nervous systems and are thought to optimize sensory computations. However, the cellular and spatial organization of interhemispheric networks and the computational properties they mediate in vertebrates are still poorly understood. Thus, it remains unclear to what extent the connectivity between left and right brain hemispheres participates in sensory processing. Here, we show that the zebrafish olfactory bulbs (OBs) receive direct interhemispheric projections from their contralateral counterparts in addition to top-down inputs from the contralateral zebrafish homolog of olfactory cortex. The direct interhemispheric projections between the OBs reach peripheral layers of the contralateral OB and retain a precise topographic organization, which directly connects similarly tuned olfactory glomeruli across hemispheres. In contrast, interhemispheric top-down inputs consist of diffuse projections that broadly innervate the inhibitory granule cell layer. Jointly, these interhemispheric connections elicit a balance of topographically organized excitation and nontopographic inhibition on the contralateral OB and modulate odor responses. We show that the interhemispheric connections in the olfactory system enable the modulation of odor response and contribute to a small but significant improvement in the detection of a reproductive pheromone when presented together with complex olfactory cues by potentiating the response of the pheromone selective neurons. Taken together, our data show a previously unknown function for an interhemispheric connection between chemosensory maps of the olfactory system.

## Abstract 2

Interhemispheric connections enable interaction and integration of sensory information in bilaterian nervous systems and are thought to optimize sensory computations. This study shows that interhemispheric olfactory connections in the zebrafish brain improve the detection of a reproductive pheromone within a noisy odor background.

## Introduction

In bilaterians, information detected by parallel sensory channels is topographically represented onto sensory regions located in mirrored positions of the brain hemispheres. Communication between bilateral sensory regions with similar functions is mediated by a dense network of fibers projecting contralaterally between brain hemispheres, which are organized into commissural tracts [1–4]. These interhemispheric connections are present in invertebrates [5,6] and vertebrates [7,8]. Theories differ about how these interhemispheric connections contribute to neural computations, in particular with regard to their inhibitory or excitatory effect [9,10]. Interhemispheric inhibition has been suggested to facilitate visual [11,12] and somatosensory processing [13,14] by increasing the perceptual threshold in the hemisphere contralateral to the stimulated hemisphere and thereby shifting attention to the relevant input. The functional diversity of interhemispheric connections is present in the auditory system, in which both inhibitory and excitatory interhemispheric interactions have been observed [15,16]. The synchronization between auditory hemispheres might support figure/ground segregation, whereas interhemispheric inhibition amplifies binaural activation delay and could underlie stereo-hearing [17]. On the other hand, information sharing between hemispheres through excitation in the primary visual cortex enables binocular vision, supporting depth perception [18,19]. It also avoids the dissociation between lateralized associative centers by transferring unilaterally learned information to the opposing hemisphere. For example, the restricted lesion of fibers linking the somatosensory cortices blocks the transfer of texture learning from one paw to the other in monkeys [20]; and disconnecting the fibers responsible for the visual interhemispheric cross talk prevents the transfer of a learned visual discrimination task from one hemifield to the other [21]. Despite extensive studies, the precise cellular-level mapping of interhemispheric projections linking bilateral sensory regions, as well as the functional properties they mediate, remains largely elusive.

In the olfactory system, the activation of receptors located in spatially segregated bilateral nostrils is transformed through ipsilateral projections into topographically organized, mirror-symmetric sensory maps in the olfactory bulbs (OBs) [22–24]. However, these segregated olfactory processing channels were also shown to exhibit prominent interhemispheric interactions in several taxa. For example, in fruit flies, sensory neurons project bilaterally to the antennal lobes [25] and were suggested to improve the signal quality by pooling multiple input channels from both antennae [26]. Rodents are able to locate an odor source using bilateral sampling [27,28], which depends on intact interhemispheric communication [27]. A multisynaptic system links the left and right OBs via the anterior olfactory nucleus (AON) [29], a circuit that was proposed to support the interhemispheric transfer of olfactory memory [30]. Beyond such connections through higher olfactory areas of vertebrates, direct projections were shown to link both OBs in teleost fish [31]. However, it is less clear how all these interhemispheric connections between olfactory processing channels across the vertebrate brain work in parallel and contribute to olfactory computations. The function of interhemispheric olfactory connections is perhaps best conceptualized when asking what could be the advantage for vertebrates to maintain redundant olfactory maps in each hemisphere. Indeed, unilateral olfactory input is sufficient to detect, identify, and spatially locate odors [32,33]. Yet bilateral sensory maps linked by interhemispheric connections could confer additional properties akin to visual depth perception [34] and precise sound source localization [35]. In line with this, prior evidence suggests that bilateral olfactory inputs improve the speed and the accuracy of odor tracking in a variety of animal species [28,32,33,36]. The neural circuits serving the improved chemotaxis are currently unknown; however, mechanisms have been proposed, including increased signal-to-noise ratio [32] or interhemispheric inhibition [37]. Alternatively, redundant information could prove essential when a sensory organ is (partially) obstructed, as can be the case in both rodents and humans [38,39]. Interhemispheric transfer of unilaterally experienced odorant information through AON would then help maintain both olfactory maps in the cortex up to date, even when one of the sensory organs is impaired [30,39,40]. Despite these studies, the precise neural circuit that links the two olfactory hemispheres, and how this circuit contributes to olfactory computations, is yet to be fully elucidated.

To address this, we used an ex vivo preparation of the adult zebrafish brain and characterized interhemispheric connections between the primary brain regions processing odor information, the OBs. First, we show that the olfactory glomeruli receive direct and spatially organized mitral cell projections from similarly tuned glomeruli at the contralateral OB. We also show that the OB interneurons receive top-down projections from the contralateral telencephalic olfactory centers. Second, we investigate the function of these interhemispheric connections by using two-photon calcium imaging in the OB. We find that interhemispheric connections elicit a balance of topographically organized odor-specific excitation and nontopographically organized inhibition on the contralateral OB. Our results reveal that strong contralaterally evoked activity can modulate odor responses in an intensity-dependent manner. Finally, we demonstrate that the excitatory and inhibitory interhemispheric interactions jointly improve the detection of a reproductive pheromone within a background of olfactory noise elicited by food odors by specifically potentiating the response of the pheromone-responding OB neurons. Taken together, our findings show a previously undescribed function for the interhemispheric olfactory connections for improving the detection of sensory stimuli within a noisy background.

## Results

### The OBs are connected through direct and indirect interhemispheric projections

**Fig 1:**
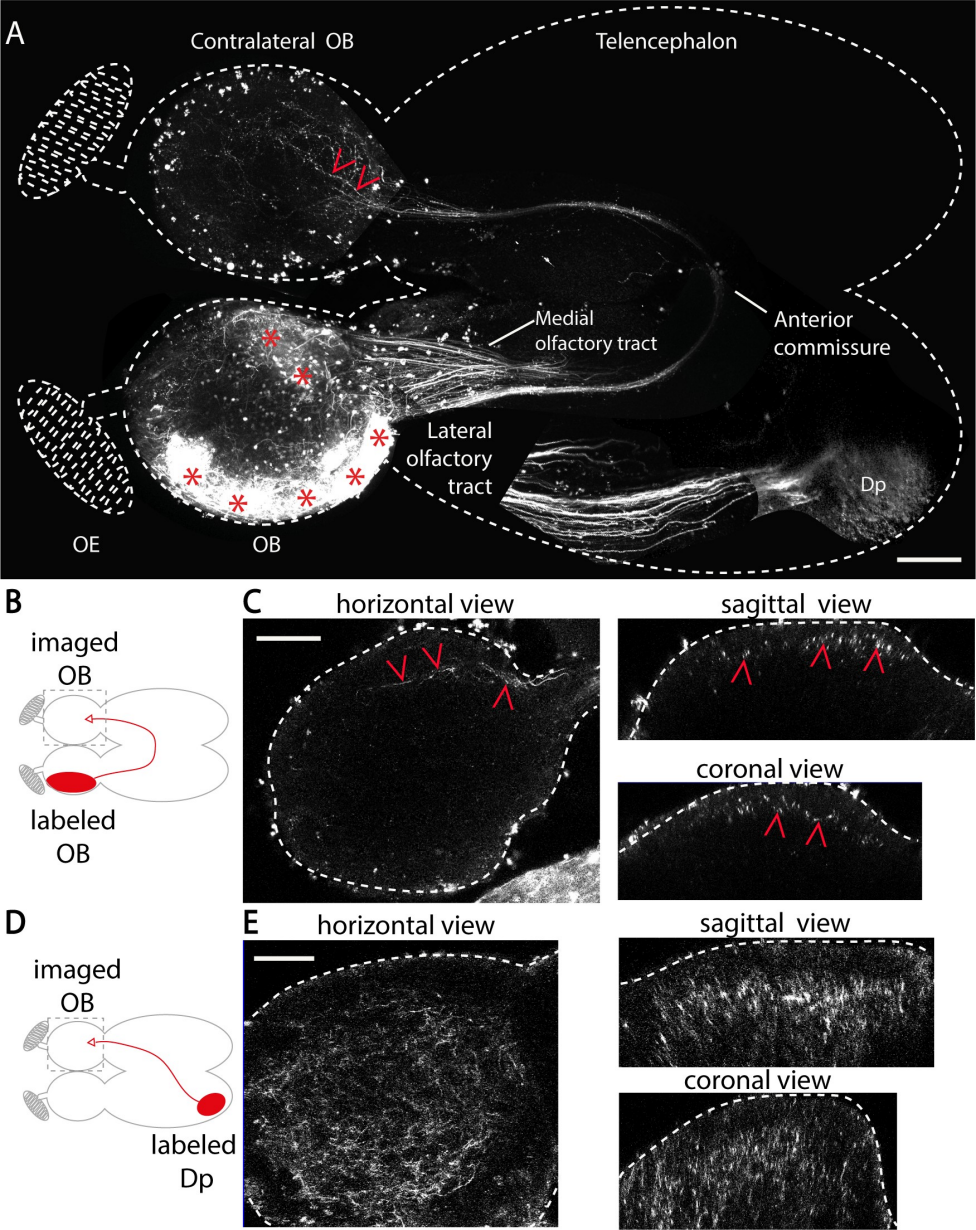
Zebrafish OBs are connected by two interhemispheric pathways. (A) Two-photon microscopy images showing unilateral dye labeling of OB cells in an adult zebrafish forebrain. This image is reconstructed by juxtaposing several partially overlapping images. Labeled mitral cell axons cross the midline at the level of the anterior commissure and terminate in the contralateral OB. Dense ipsilateral axonal projections to Dp, the fish homolog of the olfactory cortex, course through the lateral olfactory tract. Red asterisks indicate dye electroporation sites in the OB, and red arrowheads point at axonal projections in the contralateral OB. (B) Schematic of dye labeling in the OB. The filled red ellipse indicates the labeling site. The gray dashed square indicates the imaging zone in (C). (C) Horizontal, sagittal, and coronal views of mitral cell axons projecting to the contralateral OB in a representative brain (*n* = 12 fish). (D) Schematic of dye labeling of Dp. The gray dashed square indicates the imaging zone in (E). (E) Horizontal, sagittal, and coronal views of Dp projections to the contralateral OB in a representative brain (*n* = 5 fish). Scale bars represent 100 μm. Dp, dorsal part of the dorsolateral pallium; OB, olfactory bulb; OE, olfactory epithelium.

In vertebrates, olfactory information from one nostril is sent to the ipsilateral OB. It is subsequently passed on to higher olfactory areas primarily on the ipsilateral side [41]. In the current study, we used an ex vivo explant preparation of the adult zebrafish brain [22,42,43] to investigate how bilateral OBs are interconnected across the brain hemispheres. We first investigated whether mitral cells directly project to the contralateral OB (interbulbar projections). To do so, OB neurons were labeled by electroporation of tetramethylrhodamine (TMR)-dextran [44]. Two-photon microscopy was then used to image the labeling sites as well as the axonal projections to the contralateral OB (**Fig 1A and 1B**). Mitral cell axons exited the ipsilateral OB through the medial and lateral olfactory tracts and mostly terminated in the ipsilateral dorsal part of the dorsolateral pallium (Dp), which is a zebrafish telencephalic area homologous to the rodent olfactory cortex [45] (**Fig 1A**). Interestingly, we also observed that axons coursing through the medial olfactory tract crossed to the contralateral hemisphere at the level of the anterior commissure (**Fig 1A**). These axons terminated at the peripheral layers of the contralateral OB, where the glomeruli and the mitral cells are located (**Fig 1A, 1B and 1C**).

The vertebrate OB is innervated by centrifugal fibers originating from the ipsilateral telencephalon, where higher olfactory centers are located [46–49]. To investigate the extent of contralateral inputs received by the OB from higher olfactory centers, we asked whether top-down projections from Dp reached the contralateral OB in adult zebrafish. To do so, we unilaterally labeled Dp neurons using local dye electroporation (**Fig 1D**). Neural processes extending from the Dp diffusely innervated mostly the deep layers of the ipsilateral (**S1A Fig, S1B Fig**) and contralateral OB (depth > 50 μm**, Fig 1E**), where the majority of the inhibitory interneurons are located (**S2 Fig**). Although the contralateral Dp innervations mostly targeted the core layers of the OB, they were not strictly restricted to these core layers. To further characterize Dp projections to the OB, we electroporated TMR-dextran at the deep layers of the OB (see Materials and methods) and imaged the retrogradely labeled neurons in both ipsi- and contralateral Dp (**S1C Fig**, *n* = 4 fish). We found retrogradely labeled neurons both at the ipsilateral Dp (**S1D Fig**) and at the contralateral Dp (**S1E Fig**). These results confirm that the interneurons at the deep layers of the OB receive direct innervation from contralateral Dp.

Altogether, our results revealed that the zebrafish OBs are connected through at least two major types of interhemispheric connections. A direct and topographically organized mitral cell projection terminates on the peripheral layer of the contralateral OB. An indirect connection through the zebrafish homolog of the olfactory cortex, Dp, predominantly terminates at the deep layers of the contralateral OB, where most inhibitory interneurons reside.

### The direct interbulbar projections retain a fine-scale chemotopic organization

**Fig 2:**
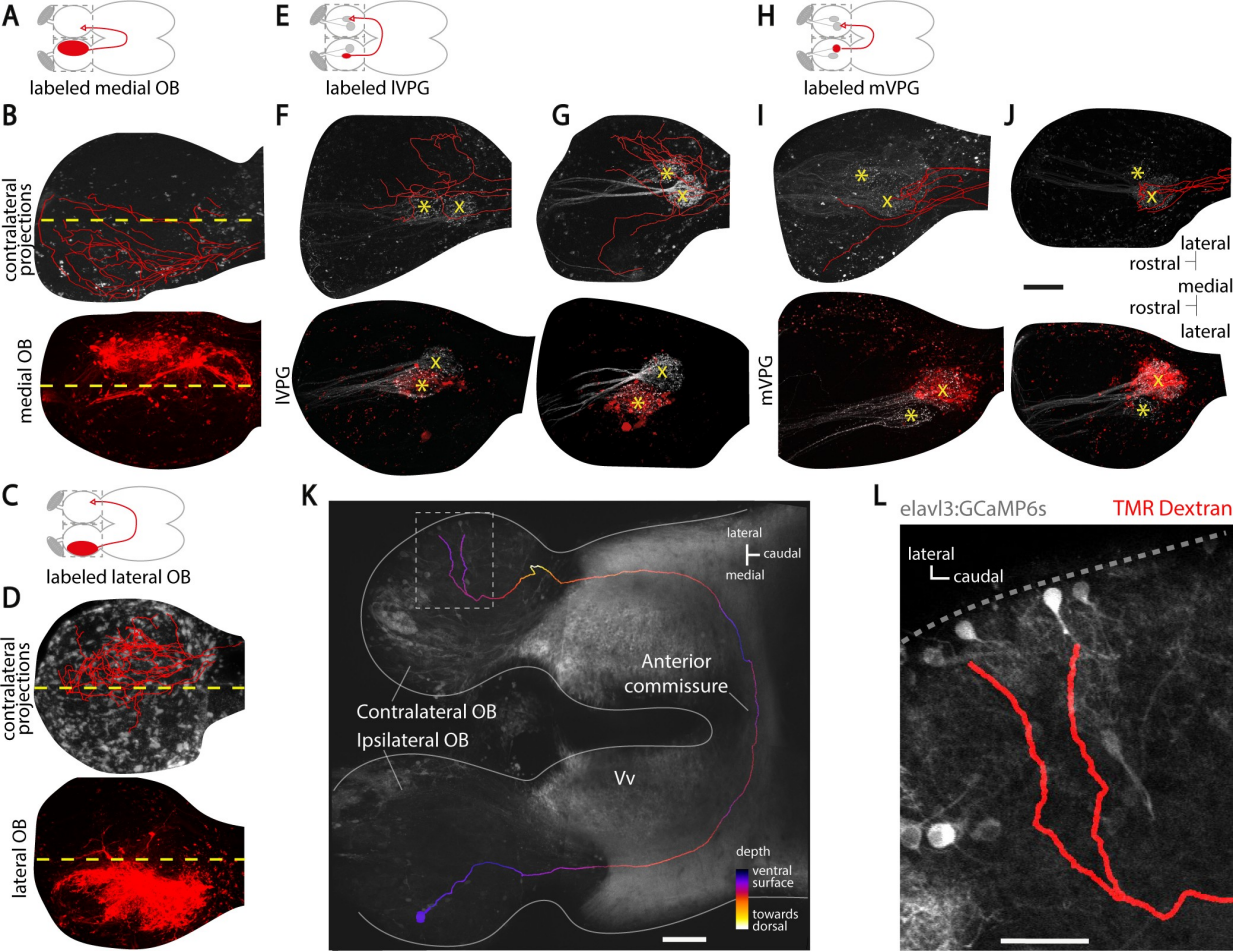
The direct interbulbar connections are topographically organized and glomeruli specific. (A) Schematic of the dye labeling in the medial OB. The filled red ellipse indicates the labeling site. The gray dashed squares indicate the two imaging fields. (B) Two-photon microscopy images showing the labeled neurons in the medial OB (bottom) and their reconstructed axonal projections (red) to the contralateral medial OB (top, *n* = 3 fish). The yellow dashed line separates each OB into a medial and a lateral half. (C) Schematic of the dye labeling in the lateral OB. (D) Two-photon microscopy images showing the labeled neurons in the lateral OB (bottom) and their reconstructed axonal projections (red) to the contralateral lateral OB (top) in a representative brain (*n* = 7 fish). (E) Schematic of the dye labeling of the lVPG. (F and G) Two-photon microscopy images showing the labeled neurons innervating the lVPG (bottom) and their reconstructed axonal projections (red) to the contralateral OB (top, *n* = 2 fish). (H). Schematic of the dye labeling of the mVPG. (I and J) Two-photon microscopy images showing the labeled neurons innervating the mVPG (bottom) and their reconstructed axonal projections (red) to the contralateral OB (top, *n* = 2 fish). The lVPG is indicated by a yellow asterisk, and the mVPG is indicated by a yellow cross. Scale bar represents 100 μm for (B–J). (K) Two-photon microscopy image of an adult zebrafish forebrain showing the reconstructed projection of a single mitral cell to the contralateral OB (*n* = 4 cells projecting to the contralateral OB, see Materials and methods). The gray background is elavl3:GCaMP6s labeling. The axonal reconstruction is color-coded for depth. The plain gray line highlights the contour of the zebrafish forebrain explant. The gray dashed square indicates the imaging zone in (B). The scale bar represents 50 μm. (L) Close-up image of the labeled mitral cell’s axonal terminals at the contralateral OB. Note the close apposition of the labeled axon with contralateral mitral cells’ dendritic tuft. The dashed gray line highlights the contour of the brain explant. The scale bar represents 25 μm. elavl3, ELAV-like RNA binding protein 3; GCaMP6, genetically encoded calcium sensor, circular permutated green florescent protein-Calmodulin-M13 peptide 6; lVPG, lateral ventroposterior glomerulus; mVPG, medial ventroposterior glomerulus; OB, olfactory bulb; TMR, tetramethylrhodamine; Vv, ventral nucleus of the ventral telencephalon.

In the zebrafish OB, odors with similar chemical properties are encoded by the activation of spatially nearby domains, forming a chemotopic map [22] that is largely stereotypical across individual animals [50,51]. For example, the neurons responding to amino acids, which are feeding cues, are located in the lateral OB [52], whereas neurons responding to bile acids, which are putative social cues, are located in the medial OB [22,52]. We therefore investigated whether mitral cell projections at the contralateral OB are topographically organized. Labeling mitral cells located in the medial OB revealed projections terminating at the medial domain of the contralateral OB (**Fig 2A and 2B**), thus mirroring their cell body location. Similarly, labeling mitral cells at the lateral OB revealed projections terminating at the lateral domain of the contralateral OB (**Fig 2C and 2D**). These results suggested that direct interbulbar projections are topographically organized based on the functional domain from which they originate.

Next, we tested whether the direct interbulbar projections can specifically link mirror-symmetric glomeruli with similar odor tuning (isofunctional glomeruli) across hemispheres. To do this, we labeled mitral cells innervating one of the two genetically identified ventroposterior glomeruli, lateral ventroposterior glomerulus (lVPG, **Fig 2E**) or medial ventroposterior glomerulus (mVPG, **Fig 2H**), which can be visualized in stereotyped locations in both OBs [52]. We found that TMR-dextran electroporation into lVPG or mVPG specifically labeled somata of OB neurons within or close to the electroporated glomerulus (**Fig 2F, 2G, 2I and 2J, S3 Fig**), highlighting the spatial specificity of the electroporation [44]. We observed that lVPG mitral cells sent interhemispheric projections that terminated next to the contralateral lVPG, as well as in nearby glomeruli (**Fig 2F and 2G**). We repeated this experiment by labeling the mitral cells of the mVPG (**Fig 2H**). We observed that the axons of mVPG mitral cells projected to the mVPG at the contralateral OB (**Fig 2I and 2J**). To further confirm that mitral cells send direct projections to the contralateral OB, we filled individual mitral cells using a patch-clamp electrode (see Materials and methods). We observed mitral cells that sent direct projections to the contralateral OB. Reconstructing the axonal projections of a filled mitral cell (**Fig 2K**) showed that it terminated at the contralateral OB, within a spatial domain similar to its soma location (**Fig 2L**), in agreement with our findings in Fig 2F and 2J. Together, these results show that direct interbulbar projections across the brain hemispheres are topographically organized and glomeruli specific.

### Interhemispheric connections between OBs modulate odor responses

**Fig 3:**
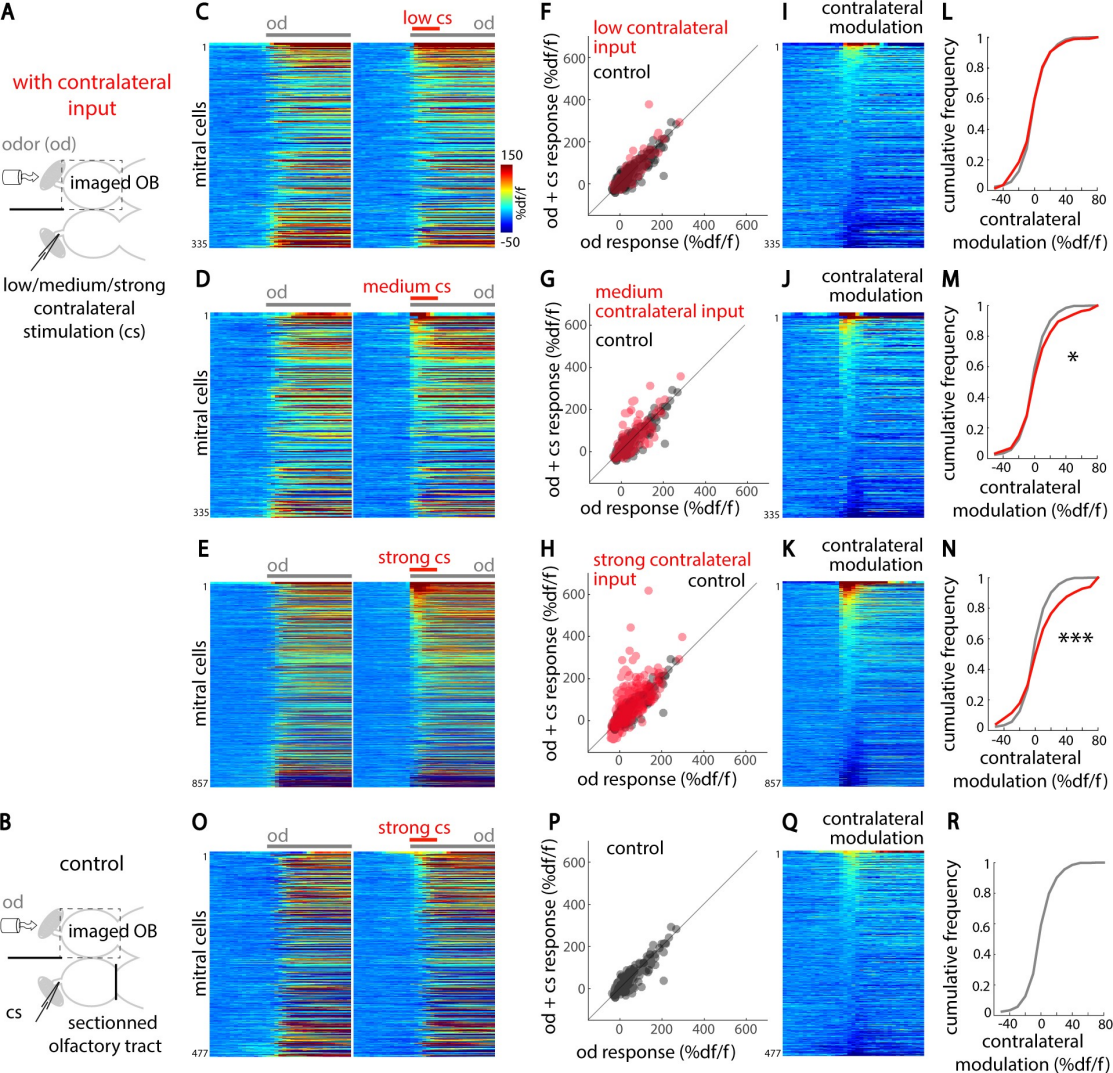
Interhemispheric connections modulate odor-evoked responses in the contralateral OB. (A) Illustration of the experimental setup depicting an adult zebrafish brain explant expressing GCaMP6s in mitral cells. A tube delivers food odor (od) selectively to the ipsilateral nostril. Contralateral stimulations (cs) of varying intensities (low, medium, and strong, see Materials and methods) are delivered using a glass microstimulation electrode inserted in the contralateral olfactory nerve. (B) The contralateral olfactory tract, which enables interhemispheric olfactory connections, is sectioned in control conditions. (C–E and O) Mean odor response time course of all mitral cells recorded in the ipsilateral OB following odor presentation (od, gray bar) or odor combined with simultaneous contralateral microelectrode stimulation (od + cs; cs indicated by a red bar) for low, medium, and strong stimulation intensities and the control condition, respectively. For each condition, mitral cells from multiple animals are pooled and sorted by the amplitude of contralateral modulation ([od + cs] − od). (F–H and P) Average mitral cells’ responses to odor (red) are plotted against their response to odor combined with cs for low, medium, and strong cs (in red) and the control condition (in black), respectively. Mitral cells above the unity line are positively modulated by contralateral inputs. (I–K and Q) The contralateral modulation ([od + cs] − od) is displayed for all mitral cells for low, medium, and strong cs and the control condition, respectively. (L–N and R) The cumulative frequency distribution of the contralateral modulation is displayed for all mitral cells for low, medium, and strong cs (in red) and the control condition (in black), respectively. The numbers of mitral cells are as follows: low cs, 335 cells in three fish; medium cs, 335 cells in three fish; strong cs, 857 cells in six fish; control cs, 477 cells in two fish (*p < 0.05; ***p < 0.001; two-sample, two-tailed Kolmogorov–Smirnov test). Numerical data used to generate this figure can be found in S1 Data and S2 Data. cs, contralateral microstimulation; GCaMP6, genetically encoded calcium sensor, circular permutated green florescent protein-Calmodulin-M13 peptide 6; OB, olfactory bulb; od, food odor extract.

The presence of these extensive interhemispheric connections raised the possibility that olfactory information in one hemisphere can influence odor processing in the contralateral OB. To test this hypothesis, we measured odor-evoked responses using two-photon calcium imaging in zebrafish expressing the genetically encoded calcium sensor, circular permutated green florescent protein-Calmodulin-M13 peptide 6 (GCaMP6s) under the ELAV-like RNA binding protein 3 (elavl3) promoter, primarily in mitral cells [42,53], while electrically stimulating the contralateral olfactory nerve at different intensities using a glass microelectrode (**Fig 3A and 3B, S4 Fig**). As expected, food odor activated numerous ipsilateral mitral cells (**Fig 3C, 3D and 3E, left panels**). Low-intensity microelectrode stimulation of the contralateral olfactory nerve led to modulation of odor response in very few ipsilateral mitral cells (**Fig 3C and 3F, right panels**). However, as we increased the contralateral stimulation to medium and strong intensities, more ipsilateral mitral cells showed increased odor responses (**Fig 3D, 3G, 3E and 3H**). Importantly, to verify that contralateral stimulation did not directly activate ipsilateral neurons through volume transmission, we repeated the same experiment after sectioning contralateral mitral cell axons at the level of the olfactory tract (**Fig 3B**). In that case, we did not observe modulation of the mitral cell responses, even when using the strongest microstimulation intensity (**Fig 3O, 3P, 3Q and 3R**), indicating the necessity of intact contralateral olfactory inputs. We next calculated the contribution of contralateral inputs to odor responses by measuring the difference of amplitude between the ipsilateral odor responses with and without the contralateral microelectrode stimulation (**Fig 3I, 3J and 3K**). We found significant and mainly excitatory modulation of odor responses by medium and strong contralateral inputs (**Fig 3M and 3N**). Our results show that interhemispheric connections across the OBs can dynamically modulate the odor response in the contralateral OB.

### Natural odors elicit odor-specific and chemotopically organized excitation in the contralateral OB

**Fig 4:**
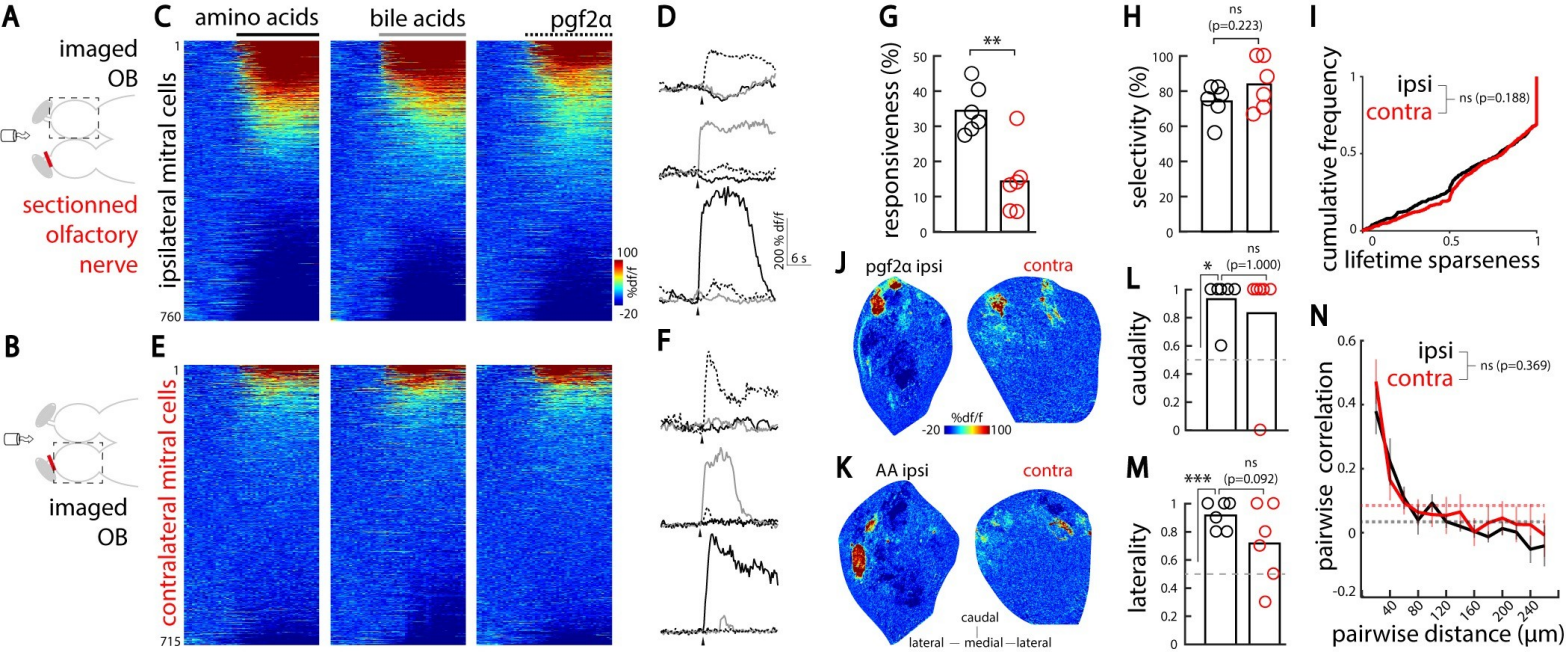
Natural odors elicit odor-selective and chemotopically organized responses in the contralateral OB. (A) Illustration of the experimental setup depicting an adult zebrafish brain explant expressing GCaMP6s in mitral cells. A tube delivers the olfactory cues to the nostrils. The stimuli used are AAs, bile acids, and pgf2α. The olfactory nerve is sectioned (red mark) on the contralateral OB. Mitral cell odor responses are recorded in the OB with an intact olfactory nerve using two-photon calcium imaging. (B) Illustration of the experimental setup depicting an adult zebrafish brain explant. Mitral cell odor responses are recorded in the deafferented OB using two-photon calcium imaging. (C) Odor response time course of all ipsilateral mitral cells pooled and sorted by their response amplitude (760 cells in six fish). (D) Odor response time course of three representative odor-selective ipsilateral mitral cells. Arrowheads indicate odor onset. (E) Odor response time course of all contralateral mitral cells pooled and sorted by decreasing responsiveness (715 cells in six fish). (F) Odor response time course of three representative odor-selective contralateral mitral cells. Arrowheads indicate odor onset. (G) The percentage of mitral cells responding to at least one olfactory cue is larger in the ipsilateral OB (black) than in the deafferented contralateral OB (red) (**p < 0.01; Student *t* test). (H) The percentage of mitral cells responding to only one of the presented odors was similar in the ipsilateral (black) and contralateral OB (red) (Student *t* test). (I) The cumulative frequency of lifetime sparseness for all mitral cells was similar in both OBs. ns; two-sample, two-tailed Kolmogorov–Smirnov test. (J) Ipsilateral (left) and contralateral (right) OB responses to pgf2α in the same fish (representative planes located 50 μm deep from the ventral OB surface). (K) Ipsilateral (left) and contralateral (right) OB responses to AA mix in the same animal than in (J) (representative planes located 90 μm deep from the ventral OB surface). (L) Spatial distribution of mitral cells responding selectively to pgf2α, along the rostrocaudal axis in both OBs for all fish. Values close to 1 indicate caudal locations, whereas values close to 0 indicate rostral locations. Note the caudal location of pgf2α-selective ipsilateral cells compared with the random distribution indicated by the gray dashed line (*p < 0.05; Mann–Whitney U test). The caudality index of contralateral pgf2α-selective cells is similar to that of ipsilateral pgf2α-selective cells (ipsi- versus contralateral, ns; Mann–Whitney U test). (M) Location of mitral cells responding selectively to AAs along the mediolateral axis in both OBs for all fish. Values close to 1 indicate lateral locations, whereas values close to 0 indicate medial locations. Note the lateral location of ipsilateral AA-selective cells compared with the random distribution indicated by the gray dashed line (***p < 0.001; one-sample Student *t* test). The laterality index of contralateral AA-selective cells is similar to that of ipsilateral AA-selective cells (ipsi- versus contralateral, ns; Student *t* test). (N) The pairwise similarity in mitral cell responses as a function of the distance between them (ipsilateral, *n* = 6 fish; contralateral, *n* = 6 fish; ns; Student *t* test). Dashed lines indicate the average correlation after shuffling the spatial locations of mitral cells. Numerical data used to generate this figure can be found in S1 Data and S2 Data. AA, amino acids; contra, contralateral; GCaMP6, genetically encoded calcium sensor, circular permutated green florescent protein-Calmodulin-M13 peptide 6; ipsi, ipsilateral; ns, nonsignificant; OB, olfactory bulb; pgf2α, prostaglandin 2α.

Having shown that contralateral olfactory nerve electric stimulation can modulate odor responses, we further investigated whether odor stimulation would also elicit activity in the contralateral OB. To assess this, we sectioned the olfactory nerve of the contralateral OB and delivered odors to the ipsilateral side with an intact olfactory nerve. By performing two-photon calcium imaging, we compared the odor-evoked mitral cell activity in the ipsilateral OB (**Fig 4A**) with the activity in the contralateral deafferented OB (**Fig 4B**) in response to amino acids, bile acids, and a reproductive fish pheromone, prostaglandin 2α (pgf2α). We observed neural responses to all odors, both in OB neurons with intact olfactory nerve (**Fig 4C and 4D**) and in deafferented OB neurons (**Fig 4E and 4F**), which showed that natural odors indeed elicit excitatory responses in the contralateral OB. As expected, there were more ipsilateral than contralateral odor-responsive mitral cells (**Fig 4G**). Importantly, no odor-induced activity was seen in bilaterally deafferented OBs (control condition, **S5 Fig**). The results showed that natural odors can elicit activity not only in the ipsilateral but also in the contralateral OB.

Our anatomical results showed that direct interhemispheric projections between the OBs follow a strict topographical organization and connect similarly tuned glomeruli across hemispheres (**Fig 2**). Moreover, different odor categories (such as amino acids, bile acids, and pgf2α) were previously shown to specifically activate spatially distinct OB domains [22]. Based on these findings, we hypothesized that interhemispheric connections would communicate odor category–specific information across spatially distinct domains between two hemispheres. To test this, we first compared the odor category specificity of the ipsilaterally and contralaterally evoked mitral cell odor responses. Consistently, we observed odor category–selective neurons present both in the ipsilateral as well as in the contralateral OB (**Fig 4D, 4F and 4H**). To quantify the response selectivity of OB neurons, we calculated the lifetime sparseness [54] of individual mitral cell odor responses. A neuron with high sparseness value responds primarily to one or a few odors, whereas a neuron with low sparseness value responds with a similar amplitude to several odors. We found no difference in lifetime sparseness of mitral cell odor responses in the ipsilateral OB and deafferented contralateral OB (**Fig 4I**), indicating a similar level of odor category selectivity between ipsi- and contralateral mitral cells. Next, we compared the spatial distribution of odor responses in the ipsilateral and deafferented contralateral OB. Based on visual inspection, odor responses appeared to be mirror-symmetric and, thus, located in functionally homologous spatial domains (**Fig 4J and 4K**). To quantify this mirror-symmetry, we mapped the location of mitral cells selectively responding to distinct odor categories. Because pgf2α activates a restricted set of neurons in the caudoventral OB [55], we calculated a caudality index (see Materials and methods) that measures the location of pgf2α-selective neurons along the anteroposterior axis. We found that both OBs exhibit similarly high caudality index for pgf2α (**Fig 4L**). Amino acids activate specifically the lateral zebrafish OB. Hence, we calculated a laterality index to quantify the mirror-symmetry of amino acid–selective neurons and found that both OBs exhibit similarly high laterality index (**Fig 4M**). To further quantify and compare the spatial distribution of ipsi- and contralaterally evoked odor responses, we calculated the pairwise similarity of mitral cell odor responses as a function of the distance between them [54]. Both in the ipsi- and contralaterally evoked odor responses, we observed that spatially nearby mitral cells within each OB exhibit similar odor response tuning, which rapidly decreased with increasing distance (**Fig 4N**). To confirm that contralaterally evoked activity is chemotopically organized, we measured odor responses in the same OB before and after sectioning its olfactory nerve. Despite the slight deformations due to deafferentation microsurgery, we could observe that direct and contralaterally evoked responses to an amino acid mix were primarily located within the same lateral OB domain (**S6A Fig and S6B Fig**). Similarly, direct and contralaterally evoked responses to a bile acid mix were located in the same anteromedial OB domain (**S6C Fig and S6D Fig**). Altogether, these results show that contralaterally evoked activity is odor specific and chemotopically organized.

### Natural odors elicit nontopographically organized responses in the inhibitory interneurons of the contralateral OB

**Fig 5:**
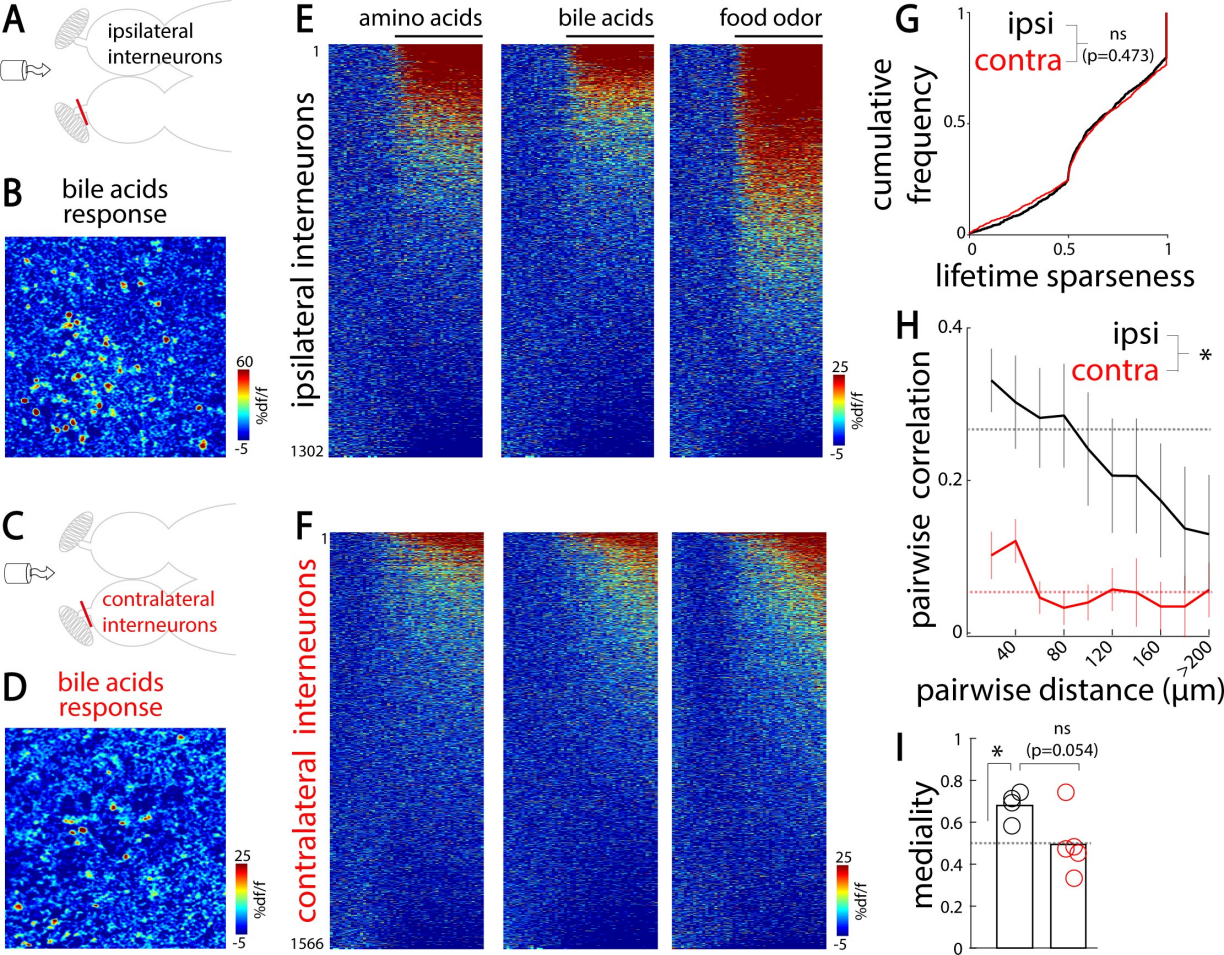
OB interneurons are diffusely activated by the contralateral olfactory inputs. (A) Illustration of the experimental setup depicting an adult zebrafish brain explant injected with a synthetic calcium indicator (Rhod-2 AM) labeling inhibitory interneurons. A tube delivers odors to the nostrils. The stimuli used are amino acids, bile acids, and food odor extract. The olfactory nerve is sectioned on the contralateral OB. (B) Ipsilateral interneurons’ response to bile acid. (C) The same setup as in (A), except that the deafferented OB contralateral to odor stimulation is imaged. (D) Contralateral interneurons’ response to bile acid. (E) Odor response time course of all ipsilateral interneurons (1,302 cells in four fish). For each odor, interneurons are sorted by decreasing responsiveness. (F) Odor response time course of all contralateral interneurons (1,566 cells in five fish). (G) Cumulative frequency of lifetime sparseness for all contralateral and ipsilateral interneurons (ns; two-sample, two-tailed Kolmogorov–Smirnov test). (H) The pairwise similarity in interneuron response as a function of the distance separating them (ipsilateral, *n* = 4 fish; contralateral, *n* = 5 fish, *p < 0.05; Student *t* test). Dashed lines indicate the average correlation after shuffling the spatial locations of interneurons. (I) Spatial distribution of ipsilateral and contralateral interneurons responding selectively to bile acids along the mediolateral axis for all fish. Values close to 1 indicate medial locations, whereas values close to 0 indicate lateral locations. Ipsilateral bile acid–selective interneurons are located medially (*p < 0.05; one-sample Student *t* test for comparison, with random distribution indicated by the gray dashed line). Contralateral bile-selective interneurons are located less medially than ipsilateral ones (*p* = 0.054, two-sample, two-tailed Student *t* test with ipsilateral interneurons). Numerical data used to generate this figure can be found in S1 Data and S2 Data. contra, contralateral; ipsi, ipsilateral; ns, nonsignificant; OB, olfactory bulb; Rhod-2 AM, acetoxymethyl ester of rhodamine-2.

Our dye electroporation of the zebrafish homolog of the olfactory cortex, Dp, showed dense projections that terminated in the granule cell layer of the contralateral OBs (**Fig 1E**), where inhibitory interneurons (granule cells) are located [56–58]. These results suggest that the two OBs are also indirectly connected through the top-down telencephalic feedbacks, likely recruiting inhibitory interneurons. To test this hypothesis, we labeled the deep layers of the OB with the synthetic calcium indicator acetoxymethyl ester of rhodamine-2 (Rhod-2 AM) and measured the odor responses of neurons located at the deep layers (>50 μm deep, [58]) of the ipsilateral (**Fig 5A and 5B**) and deafferented contralateral OBs (**Fig 5C and 5D**). Previous studies [56–58] and our anatomical recordings (**S2 Fig**) show that recording of core layers of the OB predominantly captures the activity of inhibitory interneurons because mitral cells and glomeruli are mostly absent from these regions (**S2A Fig**). Odor stimulation elicited prominent responses in the inhibitory interneurons on the ipsilateral side (**Fig 5E**). Moreover, interneurons in the deafferented contralateral OB were also activated, thus confirming our hypothesis (**Fig 5F**).

Previous studies have shown that the topographic odor map within the OB is not prominent in the rodent piriform cortex [44,59–61] and the zebrafish Dp [31,43]: axons from similarly tuned mitral cells project diffusely to these areas without strong spatial preference. Our results add to this by showing that Dp neurons in turn diffusely innervate the contralateral OB granular cell layer (**Fig 1, S1D Fig**). Based on these findings, we hypothesized that the OB interneurons’ activity pattern recruited by contralateral olfactory inputs would not be topographically organized. To test this, we first compared the odor category specificity of the ipsilaterally and contralaterally evoked interneuron responses using the lifetime sparseness measure. We observed that odor response tuning of ipsilateral and contralateral interneurons was similar (**Fig 5G**). Previous studies demonstrated that odors evoke spatially organized responses in zebrafish OB interneurons, which partially reflect the topography of mitral cell odor responses [58]. In line with these earlier results, we observed stronger correlations between odor response profiles of nearby interneurons of the ipsilateral OB (**Fig 5H**). In contrast, odor responses of interneurons in the deafferented contralateral OB exhibited significantly less spatial organization than the ipsilateral interneurons (**Fig 5H**). Similarly, we observed that chemotopically organized activation of ipsilateral interneurons elicited by bile acids was not present in the contralateral deafferented OB (**Fig 5I**). These results highlight global and nontopographic recruitment of interneurons through interhemispheric connections, which could be mediated through diffuse projections received from the zebrafish homolog of the olfactory cortex, Dp (**Fig 1E, S1E Fig**).

### Interhemispheric connections improve the detection of a reproductive pheromone within a background of olfactory noise

Our results showed that interhemispheric excitation, which originates from the contralateral OB, is spatially organized and recruits mirror-symmetric mitral cells in both OBs, whereas interhemispheric inhibition, which originates from the innervation of deeper OB layers by the contralateral Dp, is not spatially organized and recruits broadly distributed inhibitory interneurons. Such global inhibition could facilitate interhemispheric suppression and mediate odor source localization or serve as a gain control mechanism. However, strong and glomeruli-specific interhemispheric excitation could enhance the sensitivity of the OB circuit, especially for detecting odors that activate few specific glomeruli, without activating the entire network that would otherwise recruit strong global interhemispheric inhibition. Thus, we hypothesized that this balance between strong contralateral focal excitation and global inhibition could improve the detection of odors activating few glomeruli in noisy conditions, in which odor detection is challenging, for example in the presence of a background odor simultaneously activating multiple OB glomeruli.

**Fig 6:**
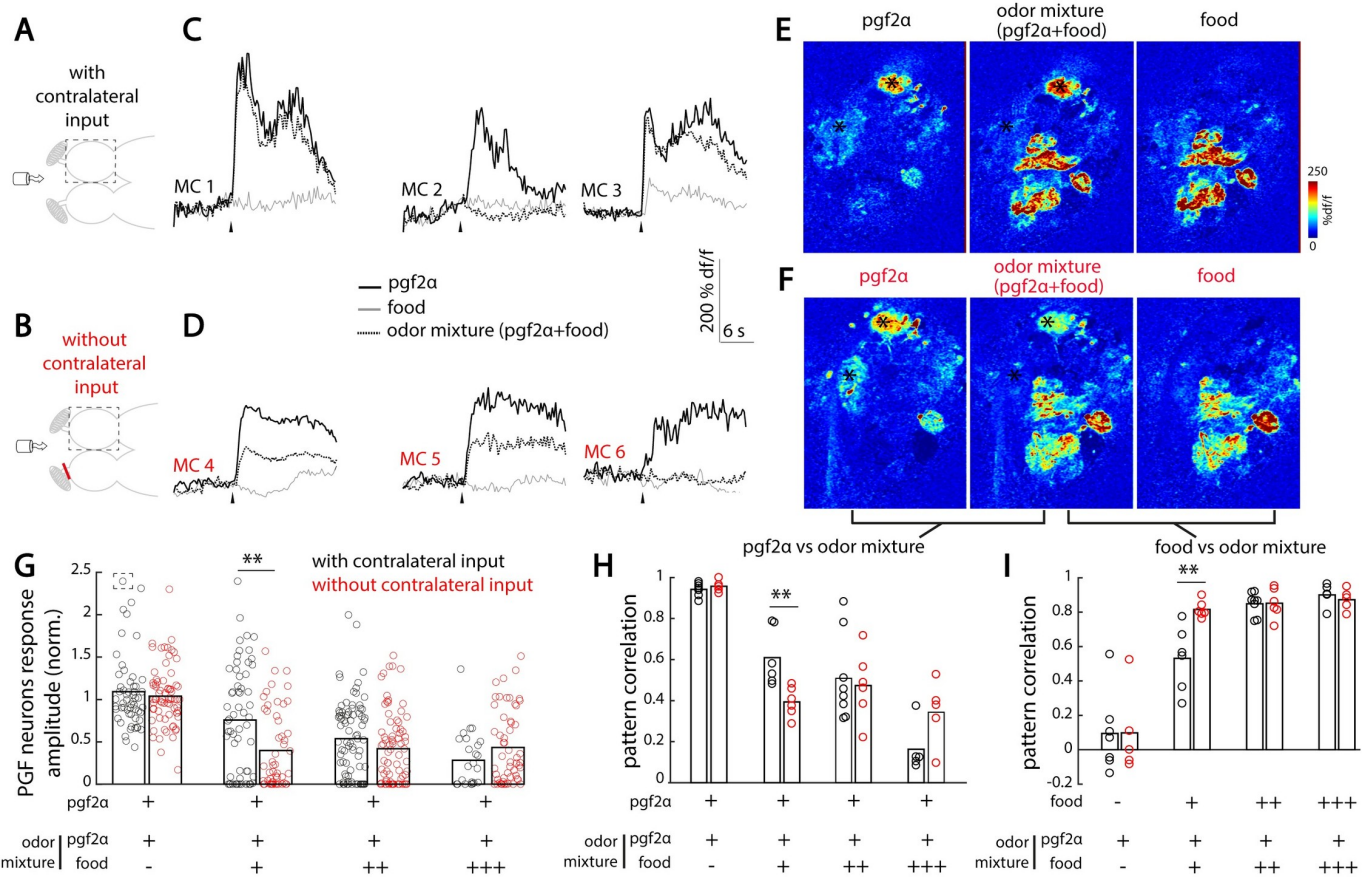
Contralateral olfactory inputs preserve reproductive pheromone detection in food odor background. (A) Illustration of the experimental setup depicting an adult zebrafish brain explant expressing GCaMP6s in excitatory neurons, and the tube for odor delivery to the intact nose. Three odors were used: pgf2α, food odor extract (food), and an odor mixture of pgf2α and food odor extract. Food odor was delivered at different concentrations (control [−]: 0 μg/mL; low [+]: 25 μg/mL; medium [++]: 100 μg/mL; high [+++]: 400 μg/mL). The dashed square indicates the imaged olfactory bulb. (B) Ventral fish brain dissection with the contralateral olfactory nerve sectioned, preventing contralateral inputs to modulate the odor responses measured. (C) Odor response time course of three representative pgf2α-selective MCs in the presence of contralateral olfactory inputs. Black arrowheads indicate odor onset. (D) Odor response time course of three representative pgf2α-selective MCs without contralateral olfactory inputs. (E) Odor response maps to pgf2α, food odor, and the odor mixture in the olfactory bulb receiving contralateral inputs. Black asterisks indicate pgf2α-selective glomeruli. (F) Odor response maps for the same fish and imaging plane than in (E), without contralateral inputs. (G) Relative response of pgf2α-selective cells to the odor mixture in all conditions. Each circle represents a pgf2α-selective MC with (black) or without (red) contralateral inputs. The response amplitude of each pgf2α-selective MC to the odor mixture is normalized to its response to pgf2α alone. Values close and superior to 1 represent conserved or enhanced responses to pgf2α in presence of food odor, respectively. Values close to 0 indicate suppression of the pgf2α response in the presence of food odor (**p < 0.01, Mann–Whitney U test). The number of pgf2α-selective MCs is as follows (with contralateral inputs): control (−), 57 MCs in seven fish; low (+), 61 MCs in six fish; medium (++), 83 MCs in eight fish; and high (+++), 25 MCs in five fish. The number of pgf2α-selective MCs is as follows (without contralateral input): control (−), 72 MCs in five fish; low (+), 53 MCs in six fish; medium (++), 77 MCs in six fish; and high (+++), 56 MCs in five fish. The response of the cell placed in a dashed square is out of range (3.3). (H) Correlation between MCs’ odor responses to pgf2α and odor mixtures for all conditions (**p < 0.01, Student *t* test). High and low pattern correlation values indicate high and low similarities with the pgf2α response pattern, respectively. Each circle represents correlation within a single fish, with contralateral input (in black) or without contralateral input (in red). (I) Correlation between MCs’ odor responses to food odor and odor mixtures for all conditions (**p < 0.01, Student *t* test). High and low pattern correlation values indicate high and low similarities with the food odor response pattern, respectively. Numerical data used to generate this figure can be found in S1 Data. GCaMP6, genetically encoded calcium sensor, circular permutated green florescent protein-Calmodulin-M13 peptide 6; MC, mitral cell; pgf2α, prostaglandin 2α.

To test the relationship between contralateral excitation and global inhibition under noisy conditions, we compared how mitral cells with or without active interhemispheric connections detect pgf2α, an olfactory cue that activates two ventral glomeruli. Pgf2α was either presented alone or together with varying concentrations of food odor, forming odor mixtures (**Fig 6A and 6B**). Our functional recordings confirmed that pgf2α activates a restricted set of selectively tuned mitral cells, whereas the food odor activates a large portion of the OB circuits (**Fig 6C, 6D, 6E and 6F**).

We observed that increasing the concentration of food odor within the odor mixtures gradually suppressed the responses of mitral cells tuned to pgf2α (**Fig 6C, 6D and 6G**). Confirming our hypothesis, we found that this suppression of pgf2α responses was less prominent in the presence of active interhemispheric connections. Indeed, pgf2α-selective mitral cells that received contralateral inputs retained 75% of their initial pgf2α response when pgf2α was presented with a low concentration of food odor, versus only 40% without contralateral inputs (**Fig 6G**). This small, but significant, improvement in pgf2α detection in the presence of food odor was likely due to the available interhemispheric excitation that compensated for the global suppression elicited by the food odor.

Next, we asked whether this improved responses of the pgf2α-selective mitral cells to the odor mixture would be sufficient to maintain pgf2α neural response pattern. Increasing the concentration of background food odor presented together with pgf2α gradually reduced the correlations between the pgf2α and mixture neural representations, shifting the mixture activity pattern away from pgf2α (**Fig 6H**) and toward food odor (**Fig 6I**). Confirming our hypothesis, we found that the presence of active interhemispheric connections counteracted the degradation of pgf2α signals in the presence of background food odors at low concentrations. Indeed, activity patterns evoked by the odor mixture were more correlated with the pgf2α alone (**Fig 6H**) and less similar to food odor (**Fig 6I**) in the presence of active interhemispheric connections. Altogether, our results show that interhemispheric communication across the OBs provides a small but significant contribution to improve the detection of a reproductive pheromone in the presence of background food odor by providing robust odor-specific excitation across isofunctional olfactory glomeruli in different brain hemispheres.

## Discussion

In the present study, we found an extensive network of interhemispheric connections between the adult zebrafish OBs with precise topography. We showed that interbulbar projections are present in adult zebrafish OB and directly connect similarly tuned olfactory glomeruli across hemispheres. A link between similarly tuned olfactory glomeruli has previously been described in fruit fly [26], in which olfactory receptor neurons send projections to olfactory glomeruli located in both hemispheres. Interestingly, these bilateral projections support rapid orientation toward the odor source through asymmetric neurotransmitter release [26]. In mice, axons of olfactory sensory neurons (OSNs) expressing the same receptor converge onto two glomeruli within the same OB, one located medially and the other laterally [23,62]. Consequently, each bulb in the mouse contains a mirror-symmetric glomerular odor map [23,63] that is absent in zebrafish, in which OSNs expressing the same receptor project onto a single glomerulus [55,64,65]. Interestingly, the murine isofunctional glomeruli within a bulb are specifically connected via an inhibitory intrabulbar circuit [66]. This circuit potentially amplifies the interglomerular activation delay in a concentration-dependent manner, leading the authors to propose that this mammalian circuit linking similarly tuned glomeruli within one bulb enables odorant concentration decoding [67]. These previous findings highlight the interspecies variability of connections linking similarly tuned glomeruli across and within brain hemispheres. Thus, it would be interesting to systematically investigate whether direct connections link the primary olfactory centers (antennal lobes or OBs) in different species. OB neurons are not known to project onto the contralateral OB in the mouse [44,68]. Yet bilaterally projecting local neurons (LNs) link both antennal lobes in fruit fly [69], and mitral cells project to the contralateral OB in teleost fish [31,47,70]. A comparative overview of the connectivity between the primary olfactory centers in species adapted to different environmental constraints will certainly provide useful insights into how these interhemispheric olfactory circuits support species-specific behaviors.

The exact role played by interhemispheric OB connections in chemosensory computations and animal behavior remains unclear. Severing the anterior commissure in mice, which prevents a broad range of interhemispheric interactions, including those between OBs, resulted in a lack of interhemispheric transfer of unilaterally formed olfactory memories [30] or loss of rapid nostril orientation toward the odor source [27]. In the present study, we found that interhemispheric olfactory connections elicited a balance of focal excitation and widespread inhibition on the contralateral OB. Such a combination of focal excitation and broad inhibition could in principle improve the detection of focal odors in a background of olfactory cues. Indeed, we found that interhemispheric connections can facilitate a small but significant improvement for the detection of a reproductive pheromone presented together with low concentrations of food odor background. This finding is particularly interesting because low concentrations of social-related olfactory cues tend to activate a restricted number of glomeruli in the primary olfactory centers of flies and fish [22,55,71] and therefore might be more sensitive to lateral inhibition in the presence of additional olfactory cues. It would be interesting to explore in further studies whether this effect also exists for other social-related odors such as bile acids [52,72,73].

In addition to the direct interbulbar projections, we found that the OBs are linked via top-down feedback originating from Dp. Top-down feedback from the piriform cortex to the ipsilateral OB has been well documented in mammals [47,74], as well as top-down feedback from Dp to the ipsilateral OB in fish [74]. Consistently, we observed top-down feedback from Dp to the ipsilateral OB, but we also found that Dp projects diffusely onto deep layers of the contralateral OB, where the inhibitory interneurons reside. In line with the lack of chemotopic organization in Dp [31,43] and the diffuse nature of Dp feedback projection to the contralateral OB, we found no obvious topographic organization of contralaterally evoked activity at the granule cell layer. This is also consistent with rodent studies showing that piriform cortex feedback mediates global and nontopographically organized inhibition in the ipsilateral OB [46,75]. It is important to note that rodent OBs are also linked via a precisely topographically organized multisynaptic circuit: the pars externa of the AON (AONpe) receives input from ipsilateral mitral cells and, in turn, projects to the contralateral inhibitory granule cell layer within the isofunctional column [29,30,40]. The contribution of these connections to the contralateral mouse OB activity was recently explored in two studies, one finding evidence of contralateral mitral cell inhibition [30], whereas another described odor-specific mitral cell excitation and found only weak and randomly distributed inhibition [40]. Thus, our study combined with previous work reveals that a similar interbulbar functional interaction, i.e., topographically organized focal excitation, potentially combined with broadly distributed inhibition, could be conserved from fish to rodents and yet mediated by a different circuit architecture.

The directly shared excitation between olfactory hemispheres that we described in this study poses the question of how fish can use an internostril delay to rapidly orient toward an odor source, as described in sharks [36]. An internostril comparison would work best with segregated and unconnected neuronal pathways that eventually converge to a brain region performing the bilateral comparison. However, the two mechanisms, i.e., interhemispheric enhancement of odor detection and bilateral comparison of segregated odor inputs, are not mutually exclusive and could coexist in the same animal. Indeed, previous research has shown that only a subset of mitral cells are linked across hemispheres. In mice, 33% of mitral cells are functionally connected to the contralateral OB [40]. In larval zebrafish, 7%–56% of the mitral cells project to the contralateral bulb, depending on the glomerulus considered [31]. Our functional results confirm this picture, with around 40% of ipsilateral activity reflected in the contralateral OB (**Fig 4G**). Thus, the remaining unconnected mitral cells could support internostril comparison for solving odor localization tasks. This could be achieved via a convergence of these bilateral unconnected cells to a higher brain region integrating inputs from both nostrils, similar to the role played by the AON in rodents [27,39].

Beyond the olfactory system, the present findings add to the existing literature describing the anatomical organization and functional role of interhemispheric connections in sensory processing [9,10]. Evidence of interhemispheric connectivity between early topographic maps in other sensory modalities than olfaction is scarce: a direct interhemispheric connection between retinae, mediated by contralaterally projecting ganglion cells, was recently identified in vertebrates [76]. This connection is only present at perinatal stages and might underlie the synchronization of bilateral retinal waves during development. As interbulbar connections are already present at fish larval stages [31], during which the olfactory maps are established and refined [77], the zebrafish interbulbar pathway might serve a similar role in maintaining the symmetry of bilateral odor maps during development. Yet the maintenance of this extensive and highly spatially organized circuit throughout adulthood, combined with its role in increased detection of an (adult) sex pheromone, suggest that this circuit is not just a remnant of embryonic structure but instead confers a substantial advantage for adult fish in detecting sensory cues important for survival.

## Materials and methods

### Experimental animal model

Experiments were conducted in adult zebrafish (*Danio rerio*) of both sexes, aged 8–14 months. The following transgenic lines were used: Tg(SAGFF179A;UAS:GFP) [52], Tg(elavl3:GCaMP6s) [53], Tg(vglut2a:DsRed) [78], Tg(gad1b:DsRed) [79], and nacre/mitfa [80]. Animals were kept under standard laboratory conditions (28.5°C; 14/10-hour light/dark cycle).

### Ethics statement

All procedures involving animals were approved and followed the animal care guidelines issued by the KULeuven Ethical Committee for animal experimentation (permit number LA1210569) and the Norwegian Food Safety Authority (permit number 19910).

### Preparations of brain explants

#### Nose-attached brain explants

The experiments were conducted in an ex vivo nose-attached brain explant preparation [22,42,43]. Adult zebrafish were euthanized by immersion in ice-cold water and then decapitated to ensure death. The head was transferred in cold artificial cerebrospinal fluid (ACSF) bubbled with carbogen (95% O_2_/5% CO_2_). The ACSF was composed of the following chemicals diluted in reverse osmosis–purified water: 131 mM NaCl, 2 mM KCl, 1.25 mM KH_2_PO_4_, 2 mM MgSO_4_7H_2_O, 10 mM glucose, 2.5 mM CaCl_2_, and 20 mM NaHCO_3_. The eyes, jaws, and ventral portions of the skull were carefully removed using forceps, exposing the ventral OBs and telencephalon and leaving the nose attached. The brain explant was then affixed using tungsten pins to a small petri dish coated with Sylgard (World Precision Instruments) and left to equilibrate at room temperature for >30 minutes under constant ACSF perfusion. Depending on the experiment, the olfactory nerve was unilaterally or bilaterally sectioned between the olfactory epithelium and the rostral end of the OB using a sharpened insect pin. Complete deafferentation was verified under a binocular microscope.

#### Bolus injection of calcium indicator

To record activity in interneurons, a synthetic calcium indicator was injected in the OB [42]. Rhod-2 AM (50 μg, Invitrogen, CAT: R1245MP) was suspended in 16 μL of dimethyl sulfoxide-pluronic (Invitrogen, CAT: P3000MP) and kept at 4°C. Aliquots of the stock solution were diluted (1/10) in ACSF the day of the experiment and gently pressure-injected using a pulled-glass micropipette. Experiments started >30 minutes after the last injection to allow for dye uptake in neurons and clearance from intercellular space.

### Olfactory stimuli

We used electrical stimulation of the olfactory nerve and bath application of odorants to the intact nose to elicit reproducible olfactory responses. For experiments with unilateral stimulation with odorants and microstimulation on the contralateral side, a small custom-made polystyrene separator was inserted in front of and below the nose to prevent the odorant to reach the contralateral nostril (**Fig 3**).

#### Olfactory nerve microstimulation

Microstimulation was performed using pulled-glass micropipettes (tip diameter approximately 10 μm) filled with ACSF and containing a silver wire connected to a computer-controlled stimulus isolator (ISO-Flex amplifier, AMPI). The tip of the micropipette was inserted in the olfactory nerve, and a train of short current pulses was applied for 3 seconds (pulse duration: 50 milliseconds; 5, 10, and 20 μA for low-, medium-, and strong-intensity stimulation, respectively).

#### Odorants

All odorants were purchased from Sigma Aldrich. Odor stocks were prepared in reverse osmosis–purified water and kept as aliquots at −20°C. Aliquots of stock solutions were diluted to their final concentration in ACSF on the day of the experiment. The amino acid mix contained arginine, asparagine, aspartic acid, alanine, phenylalanine, histidine, and methionine, each diluted at 10^−4^ mol/L. Bile acid mix contained taurodeoxycholic and taurocholic acids, each diluted at 10^−4^ mol/L. Pgf2α was used at the final concentration of 5 × 10^−7^ mol/L (similar to its estimated concentration in mature female zebrafish urine [55]), alone or mixed with food odor. Food odor stock was prepared using commercially available fish food (SDS100, Scientific Fish Food): 1 g of food was incubated in 100 mL of ACSF and filtered. Food odor stock was diluted 1/50 in ACSF for all experiments, except for the one displayed in Fig 6, in which low (dilution 1/400), medium (dilution 1/100), and high (dilution 4/100) concentrations were used. The highest food extract concentrations are similar to those eliciting robust neural [54] and behavioral [81] responses. A computer-controlled low-pressure injection valve (Valco Instruments, C22Z-3186EUH) introduced the odorants in the perfusion stream (3 mL/min) delivered through a tubing positioned 1 mm in front of the nostrils. The tubing was made of nonadherent Teflon (Teknolab, 0.8-mm internal diameter) and was rinsed with reverse osmosis–purified water after each trial to avoid contamination between successive stimuli. Multiple odorants were delivered in a pseudorandomized order and separated by at least 2 minutes to avoid neural habituation.

### Axonal projection tracing using dextran-coupled dyes

The dura covering the brain explant was carefully removed at the labeling sites using flattened-tip forceps to facilitate the dye electroporation. A solution of TMR-dextran (3,000 kDa, Invitrogen, CAT: D3306) diluted at 12 mg/mL in phosphate-buffered saline (VWR, CAT: 97062–730) was aliquoted and stored at −20°C to be thawed just before the experiment. For anterograde axonal projections imaging after labeling the OB or Dp (**Figs 1 and 2**), neurons were loaded with a fluorescent tracer using local electroporation as described in [44]. Pulled-glass micropipettes were backfilled with 2 μL of dye, and the remaining space was filled with ACSF. A silver wire was inserted in the micropipette and connected to a stimulus isolator (ISO-Flex, AMPI). The micropipette tip was directed to the center of the area of interest under a two-photon microscope, and trains of current pulses (500 pulses of 30–40 μA, 25-millisecond duration, 2 Hz) were delivered. For retrograde labeling of efferent fibers innervating interneurons’ layers of the OB, the patch pipette was inserted at the core of the OB, and the TMR dye was delivered using long current pulses (100 pulses of 1–10 μA, 6-second duration, interpulse interval = 6 seconds [82]). For labeling of single mitral cells (**Fig 2K and 2L**), a patch pipette mounted on a MultiClamp 700B amplifier headstage was loaded with two-thirds intracellular solution (in mM: 130 k-gluconate, 10 Na-gluconate, 10 HEPES, 10 Na2+-Phospho-Creatine, 4 NaCl, 4 ATP-Mg, and 0.3 Na3+-GTP) [83] and one-third TMR-dextran solution. A seal was then formed with a mitral cell, and around 25 low-intensity current pulses (20 nA; duration = 500 milliseconds) were applied to inject the dye inside the cell. The brain explant was bathed at room temperature in a flow of ACSF bubbled with carbogen for at least 6 hours to allow the dye migration along the neurites. We used a two-photon imaging system (LSM 7 MP upright with 20× water-immersion objective, Zeiss; or upright with 16× water-immersion objective, Scientifica) to take anatomical scans of the brain since it enabled access to deep structures and reduced fluorophore bleaching. A mode-locked Ti:Sapphire laser (MaiTai Spectra-Physics) tuned to 840 nm was used for combined excitation of GFP and TMR. The number of successfully filled mitral cells (37 in 12 fish) was determined by counting the number of axons exiting the OB. Labeled contralateral neurites were then reconstructed by an experimenter blind to the location of the ipsilateral electroporation sites based on the TMR labeling (red channel) in Neuron Studio [84] or using the Simple Neurite Tracer plug-in [85] in ImageJ. Neurites reconstructed were then superimposed on the stack acquired in the GFP channel that provided the OB outline. For single-cell projection tracing, a whole-brain image stack was reconstructed by merging the four stacks covering the ipsilateral and contralateral sides of the brain explant, based on the GFP channel. The axonal projection was then detected semiautomatically on the TMR channel using the Simple Neurite Tracer plug-in and overlaid on the GFP channel using the Temporal-Color Code plug-in. To map the location of OB neurons labeled by single glomerulus electroporation (**S3 Fig**), TMR-dextran-labeled somata were manually detected on the red channel using the ROI manager tool. ROIs were then superimposed on the depth projection of the corresponding GFP channel.

### Two-photon calcium imaging

Neural activity measurements were collected with two-photon laser scanning systems. For the experiment described in Fig 3, data were collected for one to three planes per fish using successive recordings of a single plane at 3.4 Hz (LSM 7 MP upright with 20× water-immersion objective, Zeiss). For the remaining experiments, volumetric images were recorded at 3.3 Hz (upright with 16× water-immersion objective, Scientifica) for six planes simultaneously, which were evenly spanning the ventral side of the OB within a range of 10–110 μm deep. In both cases, a mode-locked Ti:Sapphire laser tuned to 920 nm (for GCaMP6s recordings) or 840 nm (for Rhod-2 AM recordings) was used to excite the fluorophores.

### Calcium imaging analysis

Recordings were corrected for movement in Matlab using a modified version of the algorithm used in [83,86]. Recordings were then visually inspected and discarded whenever tissue drift was observed. Individual neurons were manually segmented, and the corresponding raw fluorescent traces were calculated by averaging the value of all pixels belonging to a given neuron for each time point. The change in fluorescence (%df/f) relative to the prestimulus baseline was calculated as follows:

$$\%df/f=\left(\frac{Ft-F0}{F0}\right)*100$$

where F_0_ is the averaged 6 seconds of prestimulus fluorescence for each neuron, and F_t_ is the fluorescence of a neuron at time *t*. The stimulus response of each neuron was calculated by averaging the %df/f during the 10-second poststimulus onset (for odor responses) or 3-second post-microstimulation onset (for contralateral olfactory nerve microstimulations). Images of stimulus responses within an entire recording plane were obtained by applying the same procedure to every pixel (instead of each neuron) and smoothening the resulting image using a Gaussian filter. To calculate the selectivity of odor response of the entire neuronal population, lifetime sparseness was calculated as follows [87,88]:

$$Lifetime\;Sparseness=\left(1-\frac{{\left(\frac{\Sigma ri}{n}\right)}^{2}}{\Sigma \frac{{ri}^{2}}{n}}\right)/(1-\frac{1}{n})$$

where *ri* is the response of an individual neuron to the *it*h odor, and *n* is the total number of odors. Cells with negative responses to all odors were not included in the calculation (remaining mitral cells: 628 ipsilateral cells in six fish and 606 contralateral cells in six fish; remaining interneurons: 1,196 ipsilateral interneurons in five fish and 1,466 contralateral interneurons in four fish). Low values indicate low selectivity, whereas values close to 1 indicate that the neuron responds to only a small fraction of the odor set tested. The thresholds for detecting a response were set to 70% df/f for mitral cells and 10% df/f for interneurons. To quantify chemotopy, the spatial location of neurons responding to a single stimulus (odor-selective neurons) was allocated to anterior versus posterior OB halves or medial versus lateral OB halves. Laterality, caudality, or mediality indices were then calculated in each animal as the ratio of neurons located in the lateral, posterior, or medial OB halves, respectively.

### Confocal anatomical imaging

Anatomical scans of the OB (**S2 Fig**) were acquired using a confocal microscope (Zeiss Examiner Z1, with 20× water-immersion objective).

### Statistical analysis

Data were examined for normality using a Shapiro–Wilk test. The significance threshold was set to *p* < 0.05. Data were then analyzed using parametric (Student *t* test) or nonparametric tests (Wilcoxon signed-rank). A two-sample, two-sided Kolmogorov–Smirnov test was used for population distribution. Data were represented as mean ± standard error of the mean unless stated otherwise.

## Supporting information

S1 Fig The granule cell layer of the OB is innervated by both ipsilateral and contralateral Dp. (A) Schematic of dye labeling of Dp. The gray dashed square indicates the imaging zone in (B). (B) Two-photon microscopy image showing centrifugal inputs from Dp to deep layers of the ipsilateral OB (associated with Fig 1E). Scale bar represents 50 μm. (C) Schematic of the dye electroporation at the granule cell layers of the OB, where interneurons are located. Dashed squares indicate the imaging fields in (D) and (E). (D) Two-photon microscopy image showing somata of retrogradely labeled neurons in the ipsilateral Dp (25 ± 13 labeled somata per fish [mean ± std], *n* = 4 fish). (E) Two-photon microscopy image showing somata of retrogradely labeled neurons in the contralateral Dp in the same fish as in (D) (12 ± 4 labeled somata per fish, *n* = 4 fish). The red arrowheads point at few labeled somata. The dashed gray lines highlight the contour of the forebrain explant. Scale bars represent 20 μm. Dp, dorsal part of the dorsolateral pallium; OB, olfactory bulb; std, standard deviation.
(TIF)

S2 Fig Distribution of excitatory and inhibitory neuron markers in the superficial and deep layers of the zebrafish OB. All images represent planes located 110 μm below the ventral surface of the OB. (A) Confocal microscopy image of the OB of a Tg(vglut2a:DsRed) adult zebrafish labeling olfactory glomeruli and mitral cell somata. (B) Confocal microscopy image of the OB of a Tg(gad1b:DsRed) adult zebrafish labeling GABAergic interneurons. (C) Raw fluorescence two-photon image of an OB injected with Rhod-2 AM in the deep layers. The inner dashed line indicates where the interneurons were detected (Fig 5). The outer dashed line indicates the outline of the OB. Scale bars represent 50 μm. Orientation is the same in all pictures as in (A). DsRed, red fluorescent protein; gad1b, glutamate decarboxylase 1; OB, olfactory bulb; Rhod-2 AM, acetoxymethyl ester of rhodamine-2; vglut2a, vesicular glutamate transporter 2.
(TIF)

S3 Fig Spatially restricted labeling of individual olfactory bulb’s neurons after electroporation of TMR-dextran into a single glomerulus. (A and B). Two-photon microscopy images showing the reconstructed location of labeled somata (red circles) after electroporation of the lVPG (highlighted by a red dashed line). The image in (A) (23 somata) is from the same animal as in Fig 2F. The image in (B) (30 somata) is from the same animal as in Fig 2G. (C and D). Two-photon microscopy images showing the reconstructed location of labeled somata (red circles) after electroporation of the mVPG (highlighted by a red dashed line). The image in (C) (27 somata) is from the same animal as in Fig 2I. The image in (D) (22 somata) is from the same animal as in Fig 2J. Scale bars represent 25 μm. Orientation is the same in all pictures as in (A). lVPG, lateral ventroposterior glomerulus; mVPG, medial ventroposterior glomerulus; TMR, tetramethylrhodamine.
(TIF)

S4 Fig Responses of OB neurons to electrical microstimulation of the ipsilateral olfactory nerve. (A) Illustration of the experimental setup. (B–D) Representative responses of OB’s neurons to electrical microstimulation of the olfactory nerve at low (B; 5 μA), medium (C; 10 μA), and strong (D; 20 μA) intensities (see Materials and methods, Fig 3). Scale bar represents 100 μm. OB, olfactory bulb.
(TIF)

S5 Fig Odor-induced activity on the contralateral OB. (A) Ipsilateral recordings: the olfactory nerve was sectioned on the side contralateral to the imaged OB. (B) Contralateral recordings: same as in (A), but responses from mitral cells in the deafferented OB contralateral to odor stimulation were recorded. (C) Control recordings: same as in (A) and (B), but with olfactory nerve sectioned bilaterally (control condition). (D–F) Cumulative probability distribution of mitral cells’ response to amino acids, bile acids, and pgf2α, respectively (760 ipsilateral cells in six animals; 715 contralateral cells in six animals; 349 control mitral cells in four animals; ***p < 0.001, *p < 0.05, ns; two-sample, two-tailed Kolmogorov–Smirnov test). Numerical data used to generate this figure can be found in S1 Data. OB, olfactory bulb; ns, nonsignificant; pgf2α: prostaglandin 2α.
(TIF)

S6 Fig Contralaterally evoked odor representations are chemotopically organized similarly to ipsilaterally evoked odor responses. Odor responses were recorded in the same OB before (left panels) and after (right panels) sectioning its ON. Response maps are projections of the odor response for all planes. (A) OB response to an amino acid mixture. (B) Same OB’s response to an amino acid mixture after the ON section. (C) OB response to bile acid mixture. (D) Same OB’s response to bile acid mixture after the ON section. The white dashed line delineates the contour of the OB. The red dashed line indicates the OB domain preferentially activated by each odor. The scale bar indicates 100 μm for all images. OB, olfactory bulb; ON, olfactory nerve.
(TIF)

S1 Data Numerical data associated with the figures. (XLSX)

S2 Data Numerical data associated with the figures. (MAT)

## Abbreviations

AON: anterior olfactory nucleus
AONpe: pars externa of the AON
Dp: dorsal part of the dorsolateral pallium
elavl3: ELAV-like RNA binding protein 3
GCaMP6: genetically encoded calcium sensor, circular permutated green florescent protein-Calmodulin-M13 peptide 6
LN: local neuron
lVPG: lateral ventroposterior glomerulus
mVPG: medial ventroposterior glomerulus
OB: olfactory bulb
OSN: olfactory sensory neuron
pgf2α: prostaglandin 2α
Rhod-2 AM: acetoxymethyl ester of rhodamine-2
TMR: tetramethylrhodamine
Vv: ventral nucleus of the ventral telencephalon
