# Supplementary figures and images for: Interhemispheric connections between olfactory bulbs improve odor detection
Source: PLoS Biol. 2020 Apr 20;18(4):e3000701. doi: 10.1371/journal.pbio.3000701 (PMC7192517; doi:10.1371/journal.pbio.3000701)

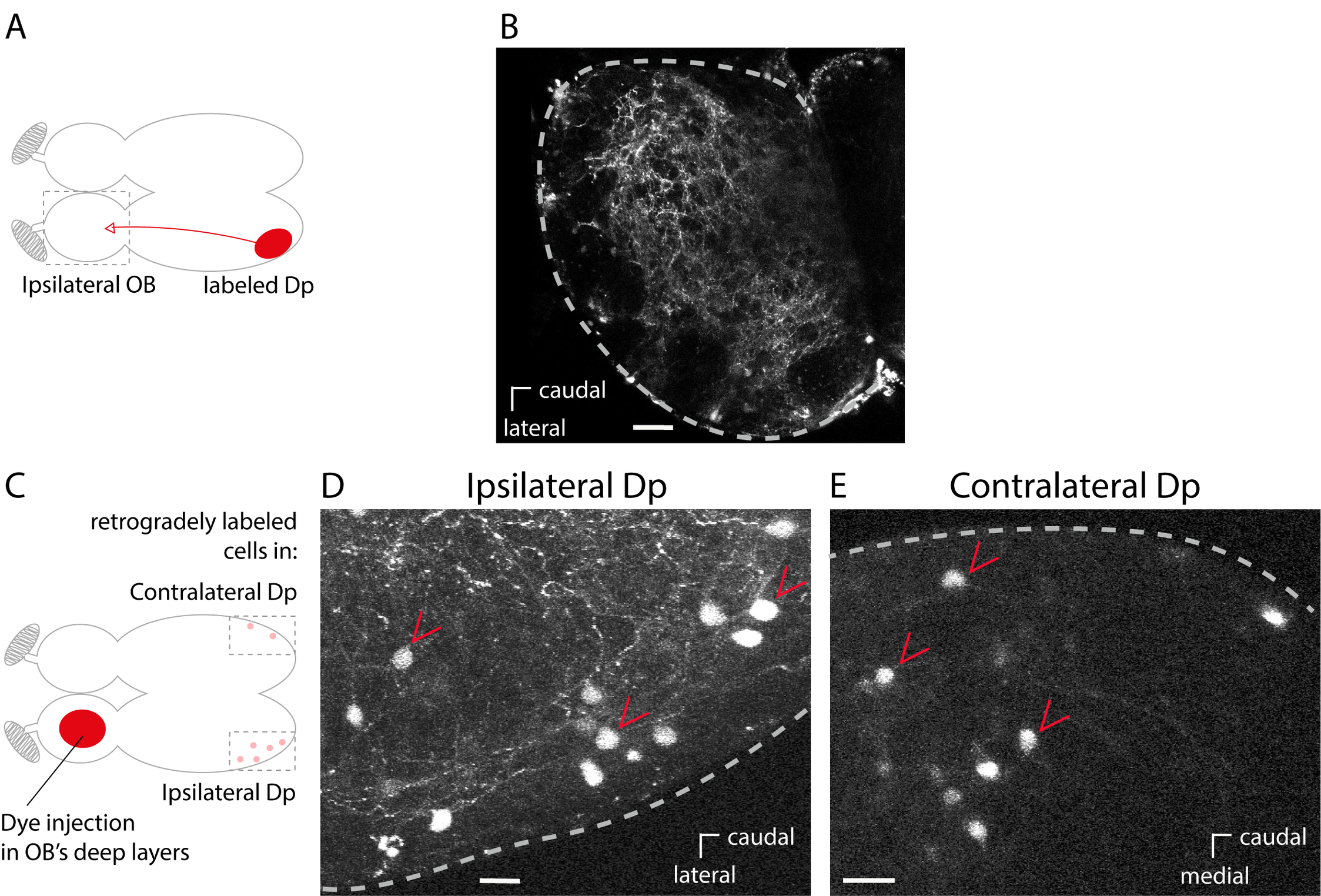

Supplement: S1 Fig — (A) Schematic of dye labeling of Dp. The gray dashed square indicates the imaging zone in (B). (B) Two-photon microscopy image showing centrifugal inputs from Dp to deep layers of the ipsilateral OB (associated with Fig 1E). Scale bar represents 50 μm. (C) Schematic of the dye electroporation at the granule cell layers of the OB, where interneurons are located. Dashed squares indicate the imaging fields in (D) and (E). (D) Two-photon microscopy image showing somata of retrogradely labeled neurons in the ipsilateral Dp (25 ± 13 labeled somata per fish [mean ± std], n = 4 fish). (E) Two-photon microscopy image showing somata of retrogradely labeled neurons in the contralateral Dp in the same fish as in (D) (12 ± 4 labeled somata per fish, n = 4 fish). The red arrowheads point at few labeled somata. The dashed gray lines highlight the contour of the forebrain explant. Scale bars represent 20 μm. Dp, dorsal part of the dorsolateral pallium; OB, olfactory bulb; std, standard deviation. (TIF) [file pbio.3000701.s001.tif]

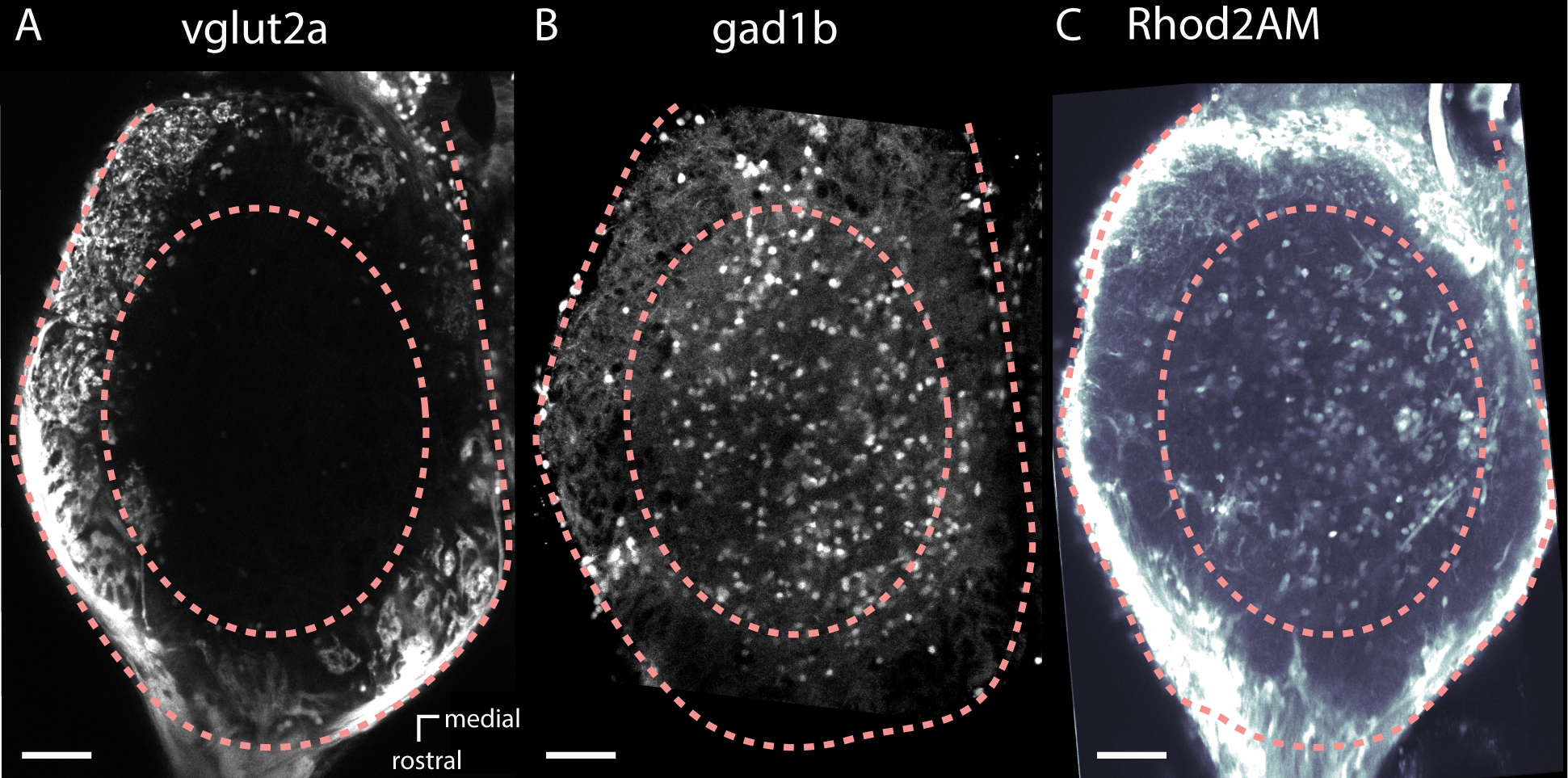

Supplement: S2 Fig — All images represent planes located 110 μm below the ventral surface of the OB. (A) Confocal microscopy image of the OB of a Tg(vglut2a:DsRed) adult zebrafish labeling olfactory glomeruli and mitral cell somata. (B) Confocal microscopy image of the OB of a Tg(gad1b:DsRed) adult zebrafish labeling GABAergic interneurons. (C) Raw fluorescence two-photon image of an OB injected with Rhod-2 AM in the deep layers. The inner dashed line indicates where the interneurons were detected (Fig 5). The outer dashed line indicates the outline of the OB. Scale bars represent 50 μm. Orientation is the same in all pictures as in (A). DsRed, red fluorescent protein; gad1b, glutamate decarboxylase 1; OB, olfactory bulb; Rhod-2 AM, acetoxymethyl ester of rhodamine-2; vglut2a, vesicular glutamate transporter 2. (TIF) [file pbio.3000701.s002.tif]

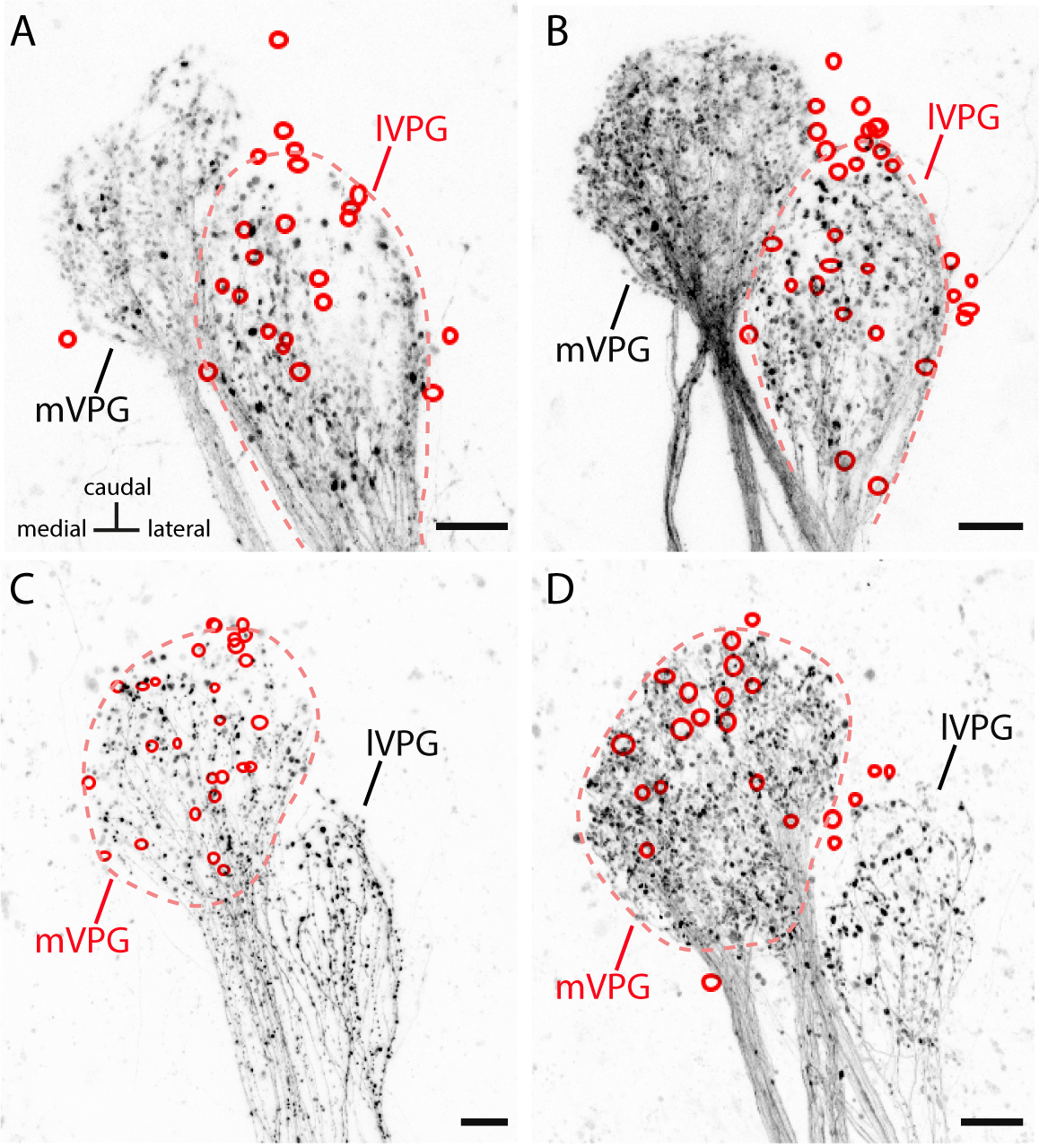

Supplement: S3 Fig — (A and B). Two-photon microscopy images showing the reconstructed location of labeled somata (red circles) after electroporation of the lVPG (highlighted by a red dashed line). The image in (A) (23 somata) is from the same animal as in Fig 2F. The image in (B) (30 somata) is from the same animal as in Fig 2G. (C and D). Two-photon microscopy images showing the reconstructed location of labeled somata (red circles) after electroporation of the mVPG (highlighted by a red dashed line). The image in (C) (27 somata) is from the same animal as in Fig 2I. The image in (D) (22 somata) is from the same animal as in Fig 2J. Scale bars represent 25 μm. Orientation is the same in all pictures as in (A). lVPG, lateral ventroposterior glomerulus; mVPG, medial ventroposterior glomerulus; TMR, tetramethylrhodamine. (TIF) [file pbio.3000701.s003.tif]

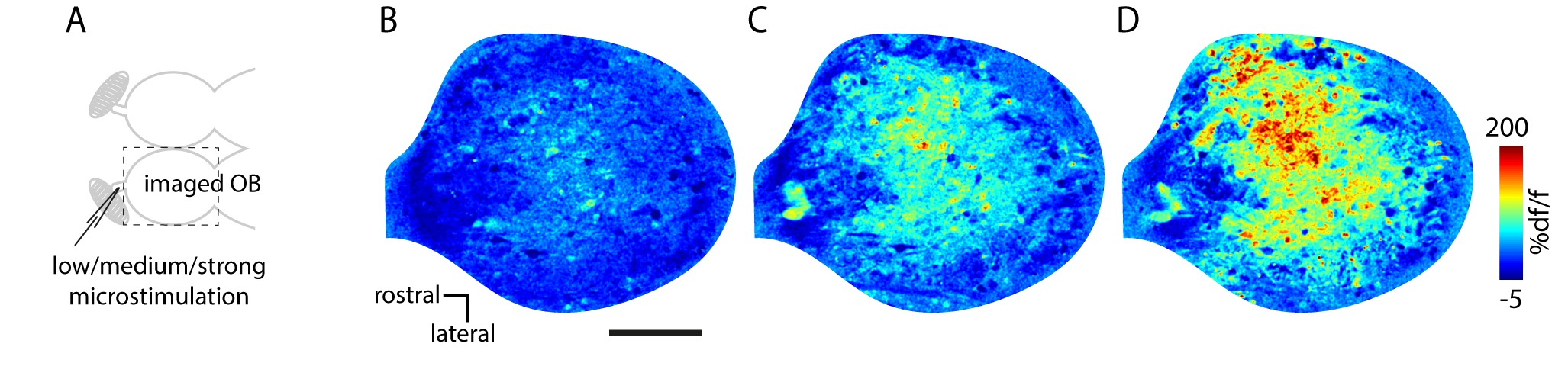

Supplement: S4 Fig — (A) Illustration of the experimental setup. (B–D) Representative responses of OB’s neurons to electrical microstimulation of the olfactory nerve at low (B; 5 μA), medium (C; 10 μA), and strong (D; 20 μA) intensities (see Materials and methods, Fig 3). Scale bar represents 100 μm. OB, olfactory bulb. (TIF) [file pbio.3000701.s004.tif]

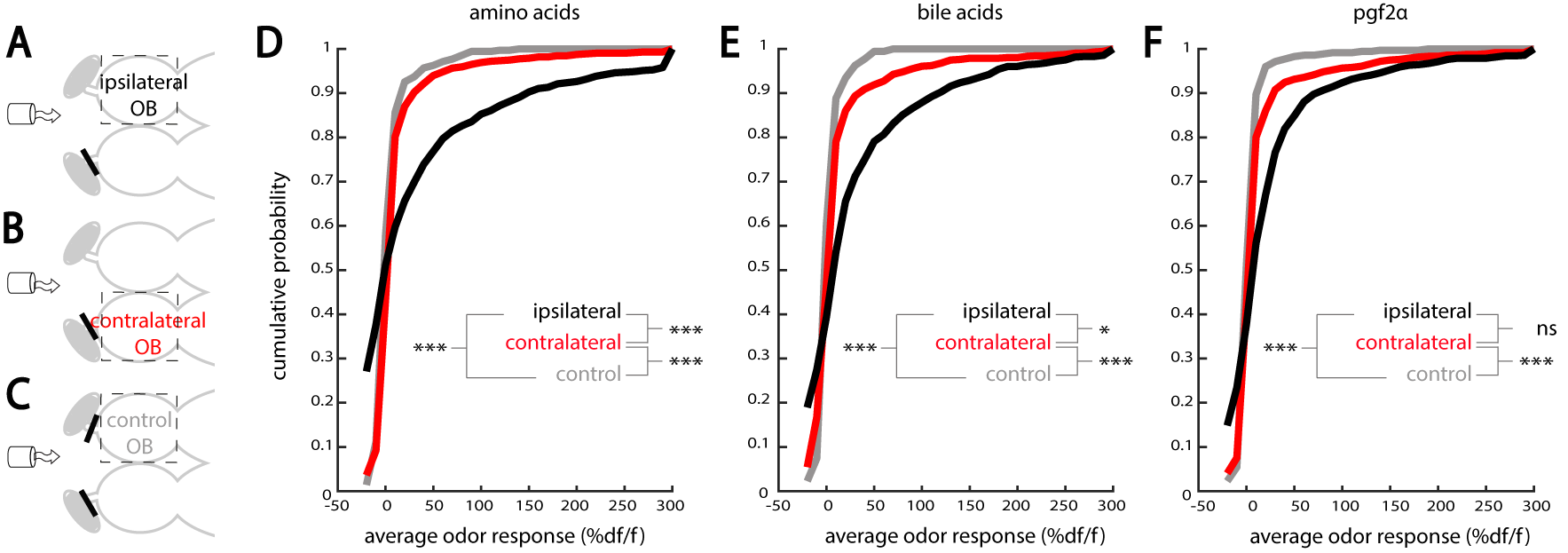

Supplement: S5 Fig — (A) Ipsilateral recordings: the olfactory nerve was sectioned on the side contralateral to the imaged OB. (B) Contralateral recordings: same as in (A), but responses from mitral cells in the deafferented OB contralateral to odor stimulation were recorded. (C) Control recordings: same as in (A) and (B), but with olfactory nerve sectioned bilaterally (control condition). (D–F) Cumulative probability distribution of mitral cells’ response to amino acids, bile acids, and pgf2α, respectively (760 ipsilateral cells in six animals; 715 contralateral cells in six animals; 349 control mitral cells in four animals; ***p < 0.001, *p < 0.05, ns; two-sample, two-tailed Kolmogorov–Smirnov test). Numerical data used to generate this figure can be found in S1 Data. OB, olfactory bulb; ns, nonsignificant; pgf2α: prostaglandin 2α. (TIF) [file pbio.3000701.s005.tif]

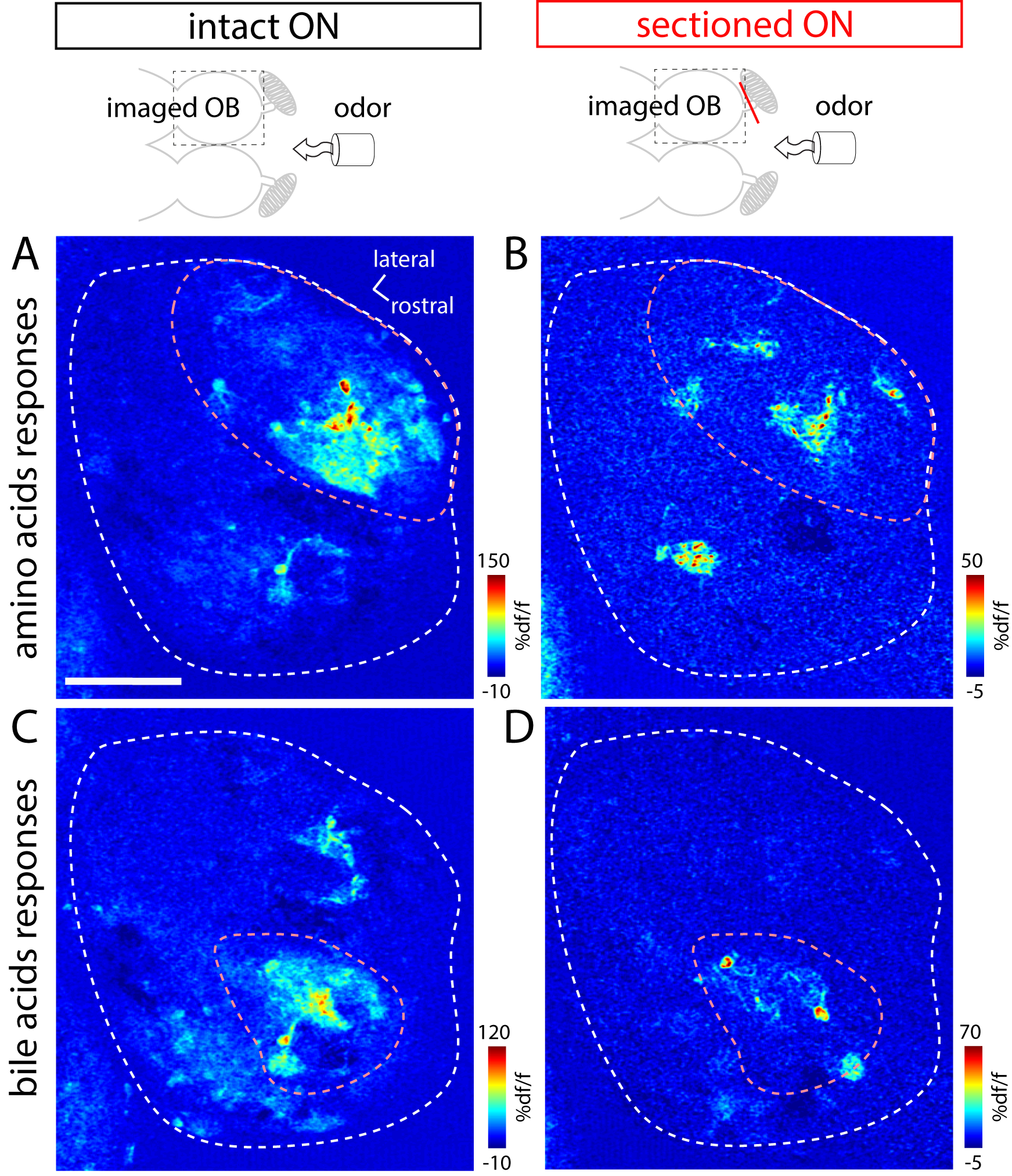

Supplement: S6 Fig — Odor responses were recorded in the same OB before (left panels) and after (right panels) sectioning its ON. Response maps are projections of the odor response for all planes. (A) OB response to an amino acid mixture. (B) Same OB’s response to an amino acid mixture after the ON section. (C) OB response to bile acid mixture. (D) Same OB’s response to bile acid mixture after the ON section. The white dashed line delineates the contour of the OB. The red dashed line indicates the OB domain preferentially activated by each odor. The scale bar indicates 100 μm for all images. OB, olfactory bulb; ON, olfactory nerve. (TIF) [file pbio.3000701.s006.tif]
